# Phenotypic and genetically predicted sleep traits in relation to dementia incidence in Swedish twins

**DOI:** 10.1002/alz.71977

**Published:** 2026-11-09

**Authors:** Manasa Shanta Näsholm, Heming Wang, Shireen Sindi, Ida K. Karlsson

**Affiliations:** ^1^ Division of Clinical Geriatrics Department of Neurobiology, Care Sciences, and Society Karolinska Institutet Stockholm Sweden; ^2^ Department of Medical Epidemiology and Biostatistics Karolinska Institutet Stockholm Sweden; ^3^ Division of Sleep and Circadian Disorders Brigham and Women's Hospital Boston Massachusetts USA; ^4^ Department of Neurology and Medicine Harvard Medical School Boston Massachusetts USA; ^5^ Ageing Epidemiology Research Unit (AGE) School of Public Health Faculty of Medicine Imperial College London London UK

**Keywords:** chronotype, composite sleep score, daytime napping, dementia, daytime sleepiness, insomnia, polygenic score, sleep duration, snoring, twins

## Abstract

**INTRODUCTION:**

Sleep and dementia are complex phenotypes shaped by genetic and environmental factors, but the genetic contribution of sleep to dementia risk remains unclear. This study examined associations of both phenotypic and genetically predicted sleep traits and their interaction with dementia risk.

**METHODS:**

Data were drawn from the Screening Across the Lifespan Twin study (1998 to 2002) within the Swedish Twin Registry. Phenotypic sleep traits included sleep duration, insomnia, snoring, daytime sleepiness, chronotype, daytime napping, and a composite sleep score. Corresponding polygenic scores (PGSs) were derived from publicly available genome‐wide association study summary statistics. Dementia diagnosis was obtained from national registers. Cox proportional hazards models estimated risk, excluding cases diagnosed within 10 years of baseline to reduce reverse causation.

**RESULTS:**

Among 13,087 individuals (mean age 70.27; 1541 dementia diagnosis cases), longer sleep duration (hazard ratio [HR] 0.93; 95% confidence interval [CI]: 0.86 to 1.00; *p* = 0.04) was associated with lower dementia risk over 13.42 years in models adjusted for sociodemographic and lifestyle behavioral factors. In within‐pair analyses, this association was attenuated. Among 16,164 genotyped individuals (mean age 55.32; 559 cases), a one‐standard deviation higher PGS for daytime napping (HR 1.12; 95% CI: 1.01 to 1.23; *p* = 0.03) was associated with higher dementia risk over 16.01 years. In 3133 individuals with both data types (mean age 66.79 years; 377 cases), this association persisted independently of phenotypic napping (HR 1.17; 95% CI: 1.03 to 1.33; *p* = 0.02), with no interaction observed.

**DISCUSSION:**

Genetic liability to daytime napping in midlife may reflect a distinct vulnerability for dementia, whereas napping in late‐life may serve a compensatory or restorative function.

## Highlights

- Longer phenotypic sleep duration was associated with a lower risk of dementia, but this association was attenuated in within‐twin pair analyses, suggesting confounding by shared genetic or familial factors.
- Phenotypic daytime napping was associated with reduced dementia risk in late life.
- In contrast, a higher PGS for daytime napping in midlife was associated with increased dementia risk, indicating opposing phenotypic and genetic associations.

## 1 BACKGROUND

Sleep and dementia are complex phenotypes influenced by both genetic and environmental factors. For late‐onset Alzheimer's disease (AD), twin studies indicate that genetic factors account for approximately 60% to 80% of disease variance^1^ and substantial heritability also for sleep traits, including sleep duration, insomnia, and sleep quality.^2^, ^3^, ^4^ Genome‐wide association studies (GWASs) further show that sleep traits are highly polygenic, with more than 600 loci identified across phenotypes.^5^, ^6^, ^7^, ^8^, ^9^, ^10^

Despite growing interest, few studies have used genetic proxies for sleep traits to examine their associations with dementia risk. Genetic approaches, such as polygenic scores (PGSs), can help disentangle biological associations from phenotypic correlations and confounding inherent in observational studies.^11^ However, evidence remains limited; a prior study reported no robust associations between PGS for sleep duration or insomnia and AD risk,^12^ and no study jointly examined phenotypic and genetically predicted sleep traits in relation to dementia risk.

Using data from the Swedish Twin Registry (STR), we examine the associations of phenotypic and genetically predicted sleep traits, as well as their interactions, with dementia risk. Within‐twin pair analyses were conducted to account for shared genetic and familial factors. We focused on five core sleep traits: sleep duration, insomnia, snoring, daytime sleepiness, and chronotype, which are widely studied and supported by well‐powered GWASs.^5^, ^6^, ^7^, ^8^, ^9^ We additionally derived a PGS for composite sleep health score,^13^ based on these traits and examined a PGS for daytime napping, a sleep trait increasingly linked to dementia risk^14^, ^15^ but not previously studied genetically in relation to dementia.

## 2 METHODS

### 2.1 Study design and participants

The data used in this study originated from the STR, the world's largest population‐based twin registry, encompassing nearly all twins born in Sweden since the late 1800s.^16^ We used data from twins born in 1958 or earlier, who participated in the Screening Across the Lifespan Twin (SALT) study conducted from 1998 to 2002 by computer‐assisted telephone interview.^17^ Of the 44,919 twin individuals in SALT, 14,388 participated in the sleep survey, of whom 14,140 remained in the final analytical sample (Figure 1).

**FIGURE 1:**
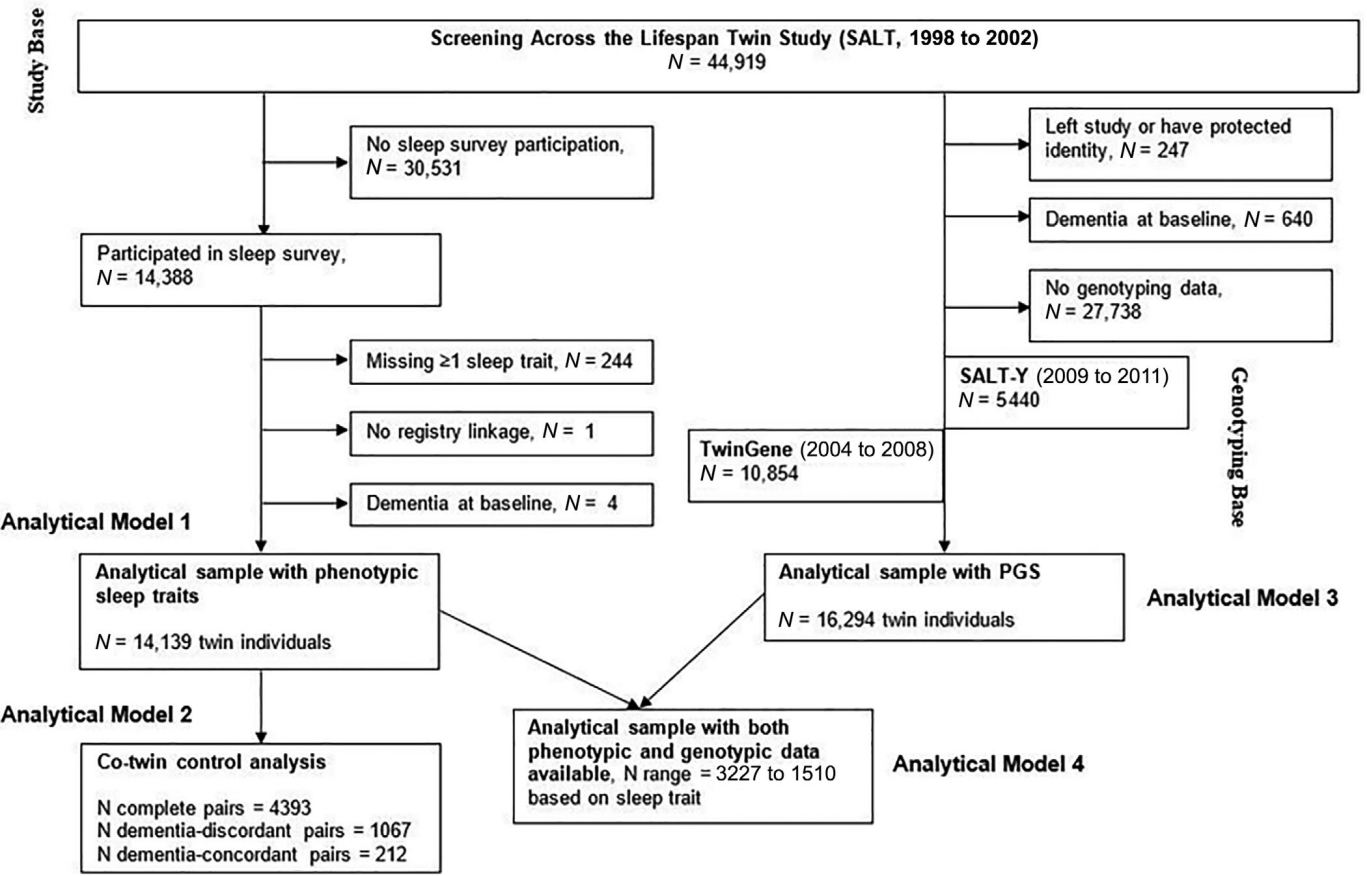
Flowchart of study participants.

Genotyping data for the construction of PGSs were available for 16,294 SALT twins (Figure 1) through participation in the substudies TwinGene (included a questionnaire, a health examination, and blood sample collection, conducted from 2004 to 2008), and Screening Across the Lifespan Twin‐Younger (SALT‐Y) (consisting of a questionnaire and saliva collection, conducted from 2009 to 2011).^17^ All participants gave informed consent, and the study was approved by the Swedish Ethical Review Authority (2024‐05625‐01).

### 2.2 Genetically predicted sleep

We used publicly available UK Biobank GWAS summary statistics for six sleep traits^5^, ^6^, ^7^, ^8^, ^9^, ^10^ and one composite sleep health score.^13^ Because the UK Biobank cohort is predominantly midlife (mean age ∼57 years), the PGSs reflect genetic predispositions for sleep traits during midlife. Full GWAS details, including sample size, questionnaire items, identified loci, and single nucleotide polymorphism (SNP) counts used for our PGS computation following quality control (QC), are provided in eTable 1 in Supplement 1.

**RESEARCH IN CONTEXT**

1. **Systematic reviews**: We reviewed the literature using PubMed. Prior studies reported mixed associations between sleep traits, particularly sleep duration and daytime napping and dementia risk, with uncertainty regarding causality and confounding by shared familial or genetic factors. PGSs have been applied to sleep traits, but no studies have combined PGSs with a twin design to jointly evaluate genetic and phenotypic sleep in relation to dementia.
2. **Interpretation**: In this Swedish twin cohort, longer phenotypic sleep duration and daytime napping in later life were associated with lower dementia risk, but attenuation in within‐twin analyses suggests shared familial or genetic confounding. In contrast, genetic liability to daytime napping was associated with higher dementia risk independent of observed behaviors, indicating a potential underlying vulnerability.
3. **Future directions**: Future work should incorporate genetic instruments that capture specific nap characteristics such as duration, timing, or frequency.

In SALT, genotyping was performed using the Human OmniExpress array (TwinGene) or the Illumina PsychArray platform (SALT‐Y), followed by standard QC and imputation against the Haplotype Reference Consortium reference panel.^18^ PGSs were constructed after standard GWAS QC,^19^ SNP filtering,^19^ linkage disequilibrium‐adjusted effect size shrinkage using SBayesR,^20^ and score computation in Plink 2.0. All PGSs were adjusted for genetic ancestry by regressing out the first five principal components and then standardized within genotyping cohort. Full details are provided in eMethods in Supplement 1.

All PGSs predicted their corresponding phenotype except daytime sleepiness (*R* ^2^ ≈ 0) in regression models adjusted for age, sex, and genotyping data collection. For the remaining traits, variance explained ranged from 1.6% (sleep health score) to 10.6% (sleep duration) (eTable 2). These values indicate modest but meaningful genetic contributions to individual differences in most sleep traits.

### 2.3 Phenotypic sleep trait assessment

A subsample of SALT participants completed the Karolinska Sleep Questionnaire (KSQ), a validated measure of subjective sleep and sleepiness.^21^ Assessed sleep traits included nighttime sleep duration, insomnia symptoms, snoring, daytime sleepiness, chronotype, and daytime napping. To harmonize phenotypic definitions with those used in the UK Biobank, KSQ measures were converted to UK Biobank‐compatible scales. An additive sleep health score was constructed based on five core traits – sleep duration, insomnia symptoms, snoring, daytime sleepiness, and chronotype – by summing dichotomized indicators of favorable sleep characteristics, yielding a total score ranging from 0 to 5, with higher scores indicating better overall sleep health. Detailed descriptions of each sleep trait, including questionnaire items, coding procedures, and harmonization steps, are provided in eMethods, with corresponding variable definitions summarized in eTable 3.

The phenotypic sleep data represent sleep patterns at the time of the baseline interview, with mean ages of approximately 70 years in the sample with phenotypic sleep measures, 55 years in the genotyped sample, and 67 years in the subsample with both phenotypic and genotypic data available. Consequently, age‐related changes in sleep traits should be considered when interpreting the relationship between genetic predisposition to sleep traits derived from a predominantly midlife population and phenotypic sleep traits measured in older adults, particularly among samples with mean baseline ages of around 70 and 67 years.

### 2.4 Outcome

Dementia diagnoses were ascertained through linkage of the STR with nationwide health registers,^17^, ^22^ including the National Patient Register (NPR), the Outpatient Register (OPR; part of NPR), the Cause of Death Register (CDR), and the Prescribed Drug Register (PDR), with follow‐up through December 31, 2016. Diagnoses were identified using International Classification of Diseases (ICD) codes for AD, vascular dementia, and other dementia as well as Anatomical Therapeutic Chemical (ATC) codes for AD medication used as diagnostic proxies.^23^ All register descriptions appear in eMethods and ICD/ATC codes in eTables 4 and 5. SALT included cognitive screening for participants aged ≥65 years,^24^ ensuring only dementia‐free individuals at baseline were included.

### 2.5 Covariates

Sociodemographic variables included education, occupational status, and marital status. Behavioral factors included alcohol risk consumption, physical activity, and smoking status. Health‐related factors consisted of current depressive symptomatology, use of hypnotics and sedatives, body mass index (BMI), prevalent diabetes, and prevalent cardiovascular diseases. Details on covariates are provided in eMethods. Apolipoprotein E (*APOE*) ε4 carrier status was defined as the presence of at least one ε4 allele, based on SNP rs429358.

### 2.6 Statistical analyses

Analyses were conducted using Cox proportional hazard regression models with age as the underlying time scale. Proportional assumption was verified by statistical testing and visual inspection of Schoenfeld's test (see eMethods and eTable 6 for details). Participants were followed from the age at phenotypic sleep trait assessment (1998 to 2002) to first record of dementia diagnosis across registers, death, or end of follow‐up (December 31, 2016), whichever occurred first. Unless otherwise stated, we accounted for relatedness among twins by applying the robust variance errors estimator for clustered observations. Individuals missing relevant data were omitted from analyses.

A series of analyses was conducted to address the specific research aims, as depicted in Figure 1. We first modeled the marginal effects of each of the phenotypic sleep traits (sleep duration, chronotype, insomnia, snoring, daytime sleepiness, daytime napping, and sleep health score) with incident dementia in the whole study population with available data on these traits (Figure 1, Analytical Model 1). To assess whether the associations between phenotypic sleep traits and incident dementia are influenced by shared genetic and familial factors, we conducted co‐twin control analyses within twin pairs (Figure 1, Analytical Model 2). In such analyses, twin individuals (within a pair) who are discordant for the outcome, or for age at onset, are compared in relation to their exposure status.^25^ As twins share both DNA (to a varying extent), in utero environment, and early life environment, the co‐twin control design elegantly controls for such confounding. In a Cox model, this is done using each twin pair as a stratum in the stratified Cox model, thereby examining the exposure–outcome association within each pair. The baseline hazard is thereby allowed to differ between twin pairs, while the resulting estimates are optimized to fit all strata and represent the within‐twin pair estimates, adjusted for factors shared between twins. If the association originally observed in the overall sample was attenuated in the co‐twin analyses, this would suggest that the relationship may be attributable to shared genetic or familial factors rather than a direct effect of the sleep trait itself.

We next examined the associations between PGSs for sleep traits and incident dementia in the full sample of genotyped individuals (Figure 1, Analytical Model 3). In the subsample with both phenotypic and genetic data available (Figure 1, Analytical Model 4), we conducted four sets of analyses: (i) marginal associations between phenotypic sleep traits and incident dementia, (ii) marginal associations between sleep PGSs and incident dementia, (iii) joint models where both the phenotypic sleep trait and its corresponding PGS were included additively to assess their mutually adjusted effects, and (iv) testing multiplicative interaction between each phenotypic sleep trait and its corresponding sleep PGS.

All regression models were adjusted for age (as the underlying timescale), sex, and study (genotyping data collection TwinGene or SALT‐Y, for PGS analyses), followed by sequential adjustment for sociodemographic, behavioral, and health‐related factors. Covariates were selected a priori based on existing literature. To minimize the potential for reverse causality, primary analyses excluded individuals who received a dementia diagnosis within 10 years of baseline, and secondary unrestricted analyses included also the first 10 years from baseline.^26^ As a post hoc analysis, we stratified analyses by *APOE* ε4 carrier status, the primary genetic risk factor for sporadic AD.^27^ To assess the potential for death to preclude the onset of dementia, we conducted sensitivity analyses using Fine‐Gray subdistribution hazard models, treating death as a competing event. These analyses were performed for the primary analyses, and the resulting subdistribution hazard ratios (SHRs) were compared with the estimates obtained from the primary Cox proportional hazards models. Co‐twin control analyses were not performed within the competing‐risk framework because Fine‐Gray models are not readily applicable to the stratified twin design used in the co‐twin analyses.

All analyses were conducted using Stata statistical software version 18.5. A two‐sided *p* < 0.05 was considered statistically significant, and the exact *p* value associated with each hazard ratio was provided to allow consideration for multiple testing. In light of the continued debate regarding the interpretation of *p* values,^28^ the results should be viewed holistically, with appropriate caution regarding potential false positive associations.

## 3 RESULTS

Baseline characteristics for the overall sample are presented in eTable 7. Participant characteristics shown for analytical samples correspond to the primary analyses, which exclude individuals with a dementia diagnosis during the first 10 years of follow‐up.

### 3.1 Analytical sample with phenotypic sleep traits

#### 3.1.1 Participant characteristics

Among 13,087 individuals with available phenotypic sleep trait data, 1541 (11.78%) were diagnosed with dementia during a mean (standard deviation [SD]) follow‐up period of 13.42 (5.34) years. The mean (SD) age at baseline was 70.27 (7.54), while the mean age at dementia diagnosis was 85.42 (5.68) years. The number of incident dementia diagnoses and the total sample size for each model adjustment are reported in eTable 8.

#### 3.1.2 Daytime napping and sleep duration are associated with incident dementia

Longer sleep duration and daytime napping, but no other sleep traits, were associated with reduced dementia risk in sex‐adjusted models (Table 1). Estimates for sleep duration were comparable when adjusting for sociodemographic, behavioral, and health‐related factors; however, the association was no longer statistically significant when adjusting for health‐related factors, possibly due to reduced sample size in the fully adjusted model. For daytime napping, the association was robust to adjustment for sociodemographic factors but fully attenuated following adjustment for behavioral and health‐related factors. In within‐twin pair analyses (Figure 1, Analytical Model 2), the associations for sleep duration (hazard ratio [HR] for sex‐adjusted model 1.03; 95% confidence interval [CI]: 0.90 to 1.19) and daytime napping (HR for sex‐adjusted model 1.02; 95% CI: 0.78 to 1.33) were fully attenuated (eTable 9 in Supplement 1).

**TABLE 1:** Association of phenotypic sleep traits with incident dementia in total sample with available self‐reported sleep traits excluding dementia cases occurring within the first 10 years of follow‐up.

	Adjusted for sex		Additionally adjusted for sociodemographic factors^a^		Additionally adjusted for behavioral factors^b^		Additionally adjusted for health‐related factors^c^	
Phenotypic traits	HR (95% CI)	*p*	HR (95% CI)	*p*	HR (95% CI)	*p*	HR (95% CI)	*p*
Sleep duration	**0.90 (0.86 to 0.94)**	**<0.0001**	**0.90 (0.85 to 0.94)**	**<0.0001**	**0.93 (0.86 to 1.00)**	**0.04**	0.93 (0.86 **to** 1.01)	0.07
Chronotype	1.02 (0.99 **to** 1.06)	0.23	1.02 (0.98 **to** 1.06)	0.34	1.01 (0.96 **to** 1.06)	0.78	1.01 (0.96 **to** 1.06)	0.73
Insomnia	0.97 (0.83 to 1.14)	0.73	1.01 (0.85 to 1.21)	0.88	1.09 (0.84 to 1.42)	0.52	1.05 (0.79 to 1.39)	0.74
Snoring	1.14 (0.99 to 1.32)	0.07	1.10 (0.94 to 1.28)	0.24	1.12 (0.90 to 1.40)	0.32	1.20 (0.95 to 1.51)	0.14
Daytime sleepiness	0.92 (0.82 to 1.03)	0.13	0.96 (0.85 to 1.08)	0.49	1.05 (0.89 to 1.25)	0.54	1.05 (0.89 to 1.25)	0.54
Daytime napping	**0.87 (0.79 to 0.96)**	**0.01**	**0.88 (0.79 to 0.98)**	**0.02**	1.03 (0.89 to 1.20)	0.71	1.03 (0.88 to 1.21)	0.67
Sleep health score	1.03 (0.95 to 1.10)	0.48	1.01 (0.93 to 1.09)	0.86	0.99 (0.88 to 1.10)	0.81	0.98 (0.87 to 1.09)	0.66

*Note*: Bold cells denote *p* < 0.05.

^a^ Sociodemographic factors include education, occupational status, and marital status.

^b^ Behavioral factors consist of alcohol risk consumption, physical activity, and smoking status.

^c^ Health‐related factors include current depressive symptomatology, use of hypnotics and sedatives, body mass index, prevalent diabetes, and prevalent cardiovascular diseases.

In secondary unrestricted analyses, not excluding dementia diagnosis within the first 10 years of follow‐up (eTable 10), sleep duration and daytime napping showed no associations with dementia risk. Insomnia was associated with a lower risk of dementia, and the within‐pair estimates were comparable to the cohort‐level findings (eTable 11).

### 3.2 Analytical sample with PGS

#### 3.2.1 Participant characteristics

Among 16,164 participants with available genotyping data for constructing PGS for sleep traits, 559 individuals (3.46%) were diagnosed with dementia over a mean (SD) follow‐up period of 16.01 (1.89) years. The mean age at baseline was 55.32 (7.94) years (range, 41 to 87 years), while the mean age at dementia diagnosis was 80.59 (7.09) years.

#### 3.2.2 PGS for midlife daytime napping is linked to greater dementia incidence

Among the PGSs for midlife sleep traits examined, only that for daytime napping showed a statistically significant association with dementia incidence (Table 2). A 1‐SD higher PGS for daytime napping was associated with an 11% (3% to 21%) higher risk of dementia in the model adjusted for sex and study, with association remaining consistent across further adjustments. Unrestricted analyses (eTable 12) yielded comparable findings.

**TABLE 2:** Association of PGS for midlife sleep traits and dementia risk in the genotyped sample excluding dementia cases occurring within the first 10 years of follow‐up.

Polygenic score (z‐score)	Adjusted for sex and study^a^		Additionally adjusted for sociodemographic factors^b^		Additionally adjusted for behavioral factors^c^		Additionally adjusted for health‐related factors^d^	
	HR (95% CI)	*p*	HR (95% CI)	*p*	HR (95% CI)	*p*	HR (95% CI)	*p*
Sleep duration	1.04 (0.95 to 1.14)	0.40	1.07 (0.97 to 1.17)	0.18	1.09 (0.98 to 1.21)	0.11	1.08 (0.97 to 1.20)	0.16
Chronotype	1.05 (0.96 to 1.15)	0.30	1.03 (0.94 to 1.14)	0.48	1.00 (0.90 to 1.12)	0.93	1.03 (0.92 to 1.15)	0.61
Insomnia	0.96 (0.88 to 1.04)	0.32	0.96 (0.88 to 1.04)	0.30	0.94 (0.85 to 1.04)	0.26	0.96 (0.87 to 1.07)	0.50
Snoring	1.02 (0.94 to 1.10)	0.69	1.02 (0.94 to 1.11)	0.69	1.01 (0.91 to 1.11)	0.87	1.00 (0.90 to 1.11)	1.00
Daytime sleepiness	1.05 (0.96 to 1.14)	0.28	1.05 (0.96 to 1.14)	0.30	1.07 (0.97 to 1.18)	0.20	1.08 (0.97 to 1.20)	0.18
Daytime napping	**1.11 (1.03** to **1.21)**	**0.01**	**1.13 (1.04** to **1.23)**	**0.005**	**1.12 (1.02** to **1.24)**	**0.02**	**1.12 (1.01** to **1.23)**	**0.03**
Sleep health score	1.01 (0.92 to 1.09)	0.90	1.01 (0.92 to 1.10)	0.90	1.01 (0.92 to 1.11)	0.84	1.01 (0.92 to 1.12)	0.79

*Note*: Bold cells denote *p* < 0.05.

^a^ Sociodemographic factors include education, occupational status, and marital status.

^b^ Behavioral factors consist of alcohol risk consumption, physical activity, and smoking status.

^c^ Health‐related factors include current depressive symptomatology, use of hypnotics and sedatives, body mass index, prevalent diabetes, and prevalent cardiovascular diseases.

### 3.3 Analytical sample with both phenotypic and genotypic data available

#### 3.3.1 Participant characteristics

A total of 3133 participants had available data on at least one sleep trait and corresponding genotyping information for constructing PGS. Among them, 377 (12.03%) individuals were diagnosed with dementia over a mean (SD) follow‐up period of 16.14 (3.12) years. The mean (SD) age of the subsample was 66.79 (6.31) years, and the mean (SD) age at dementia diagnosis was 84.02 (5.30) years.

#### 3.3.2 PGS for midlife daytime napping is associated with greater dementia risk independent of current napping habit

In the marginal models, none of the associations between phenotypic sleep traits and dementia risk were statistically significant, although the estimates for sleep duration were comparable to those observed in the full analytical sample with phenotypic sleep traits (Table 3). Among the PGSs, the PGS for daytime napping showed a statistically significant association also in this subsample. In the joint‐effect models, the association between the PGS for daytime napping and dementia risk closely mirrored the marginal estimates across all levels of adjustment, supporting a robust contribution of genetic susceptibility for daytime napping to dementia risk, also after adjusting for phenotypic daytime napping. Unrestricted analyses including diagnoses during the first 10 years of follow‐up (eTable 13) yielded similar results for the daytime napping PGS.

**TABLE 3:** Marginal and joint effect associations of phenotypic sleep traits and their corresponding midlife PGS with dementia risk excluding dementia cases occurring within the first 10 years of follow‐up.

Analytical models	Adjusted for sex and study^a^		Additionally adjusted for sociodemographic factors^b^		Additionally adjusted for behavioral factors^c^		Additionally adjusted for health‐related factors^d^	
	HR (95% CI)	*p*	HR (95% CI)	*p*	HR (95% CI)	*p*	HR (95% CI)	*p*
**Marginal effect model**								
Phenotypic trait								
Sleep duration	0.94 (0.84 to 1.04)	0.24	0.93 (0.83 to 1.04)	0.20	0.86 (0.74 to 0.99)	0.03	0.89 (0.76 to 1.03)	0.12
Chronotype	1.03 (0.96 to 1.11)	0.44	1.01 (0.94 to 1.09)	0.71	0.98 (0.90 to 1.08)	0.71	0.99 (0.90 to 1.09)	0.92
Insomnia	1.14 (0.81 to 1.62)	0.45	1.20 (0.84 to 1.72)	0.32	1.29 (0.80 to 2.08)	0.30	1.21 (0.72 to 2.03)	0.47
Snoring	1.06 (0.78 to 1.44)	0.72	1.05 (0.76 to 1.43)	0.77	0.97 (0.65 to 1.46)	0.90	1.00 (0.65 to 1.53)	0.99
Daytime sleepiness	1.06 (0.83 to 1.34)	0.66	1.07 (0.84 to 1.36)	0.59	1.28 (0.93 to 1.75)	0.13	1.27 (0.92 to 1.74)	0.15
Daytime napping	1.04 (0.85 to 1.27)	0.68	1.05 (0.85 to 1.30)	0.64	1.08 (0.83 to 1.41)	0.55	1.07 (0.81 to 1.40)	0.63
Sleep health score	0.99 (0.85 to 1.16)	0.89	0.97 (0.83 to 1.14)	0.74	1.00 (0.80 to 1.24)	0.97	1.00 (0.81 to 1.25)	0.98
Polygenic score (z‐score)								
Sleep duration	1.02 (0.92 to 1.13)	0.73	1.04 (0.93 to 1.16)	0.49	1.10 (0.96 to 1.25)	0.16	1.11 (0.97 to 1.28)	0.11
Chronotype	1.10 (0.99 to 1.22)	0.09	1.07 (0.96 to 1.20)	0.20	1.03 (0.90 to 1.17)	0.71	1.05 (0.92 to 1.21)	0.45
Insomnia	0.95 (0.83 to 1.09)	0.48	0.95 (0.83 to 1.10)	0.49	0.94 (0.79 to 1.12)	0.51	0.92 (0.76 to 1.11)	0.37
Snoring	1.07 (0.92 to 1.25)	0.39	1.11 (0.95 to 1.30)	0.19	1.06 (0.87 to 1.30)	0.55	1.05 (0.86 to 1.29)	0.62
Daytime sleepiness	1.04 (0.91 to 1.20)	0.55	1.07 (0.93 to 1.23)	0.36	1.16 (0.96 to 1.39)	0.12	1.14 (0.94 to 1.37)	0.18
Daytime napping	**1.13 (1.03 to 1.24)**	**0.01**	**1.15 (1.04 to 1.28)**	**0.01**	**1.18 (1.04 to 1.34)**	**0.01**	**1.17 (1.03 to 1.33)**	**0.02**
Sleep health score	0.98 (0.84 to 1.15)	0.82	0.95 (0.81 to 1.12)	0.55	0.95 (0.79 to 1.14)	0.57	0.95 (0.79 to 1.15)	0.60
**Joint effect model**								
Phenotypic trait								
Sleep duration	0.94 (0.84 to 1.05)	0.24	0.93 (0.83 to 1.04)	0.19	0.85 (0.74 to 0.99)	0.03	0.88 (0.76 to 1.03)	0.11
Chronotype	1.01 (0.94 to 1.09)	0.71	1.00 (0.93 to 1.08)	0.96	0.98 (0.89 to 1.08)	0.65	0.99 (0.89 to 1.09)	0.78
Insomnia	1.15 (0.81 to 1.63)	0.44	1.20 (0.84 to 1.73)	0.32	1.31 (0.81 to 2.11)	0.28	1.23 (0.73 to 2.07)	0.44
Snoring	1.03 (0.76 to 1.41)	0.83	1.01 (0.74 to 1.39)	0.94	0.98 (0.65 to 1.47)	0.92	1.00 (0.65 to 1.55)	0.99
Daytime sleepiness	1.06 (0.83 to 1.34)	0.65	1.07 (0.84 to 1.36)	0.58	1.26 (0.92 to 1.73)	0.15	1.24 (0.91 to 1.71)	0.18
Daytime napping	1.01 (0.82 to 1.23)	0.94	1.01 (0.82 to 1.25)	0.91	1.04 (0.80 to 1.35)	0.77	1.02 (0.78 to 1.34)	0.89
Sleep health score	0.99 (0.85 to 1.16)	0.90	0.98 (0.84 to 1.14)	0.77	1.00 (0.80 to 1.24)	1.00	1.01 (0.81 to 1.25)	0.95
Polygenic score (z‐score)								
Sleep duration	1.02 (0.92 to 1.14)	0.68	1.05 (0.93 to 1.17)	0.44	1.11 (0.97 to 1.26)	0.13	1.13 (0.98 to 1.29)	0.08
Chronotype	1.09 (0.98 to 1.22)	0.12	1.07 (0.96 to 1.21)	0.22	1.03 (0.90 to 1.18)	0.68	1.06 (0.92 to 1.21)	0.45
Insomnia	0.95 (0.83 to 1.09)	0.47	0.95 (0.83 to 1.10)	0.48	0.93 (0.78 to 1.12)	0.45	0.92 (0.76 to 1.10)	0.35
Snoring	1.07 (0.91 to 1.25)	0.41	1.11 (0.95 to 1.31)	0.19	1.08 (0.88 to 1.31)	0.92	1.06 (0.87—1.30)	0.54
Daytime sleepiness	1.04 (0.91 to 1.20)	0.55	1.07 (0.93 to 1.23)	0.36	1.14 (0.95 to 1.37)	0.16	1.12 (0.93 to 1.35)	0.24
Daytime napping	**1.13 (1.03 to 1.25)**	**0.01**	**1.15 (1.04 to 1.27)**	**0.01**	**1.18 (1.04 to 1.34)**	**0.01**	**1.18 (1.03 to 1.34)**	**0.01**
Sleep health score	0.98 (0.84 to 1.15)	0.83	0.95 (0.81 to 1.12)	0.56	0.95 (0.79 to 1.14)	0.57	0.95 (0.78 to 1.15)	0.59

*Note*: Bold cells denote *p* < 0.05.

^a^ For independent association of phenotypic sleep traits, model was adjusted only for sex.

^b^ Sociodemographic factors include education, occupational status, and marital status.

^c^ Behavioral factors consist of alcohol risk consumption, physical activity, and smoking status.

^d^ Health‐related factors include current depressive symptomatology, use of hypnotics and sedatives, body mass index, prevalent diabetes, and prevalent cardiovascular diseases.

Given that the PGS for daytime napping demonstrated the most robust associations across analyses, a forest plot illustrating both the phenotypic and PGS findings across analytical models and adjustment sets is provided in Figure 2. This plot reflects results from our primary analytical framework, which excludes dementia diagnoses occurring within the first 10 years of follow‐up.

**FIGURE 2:**
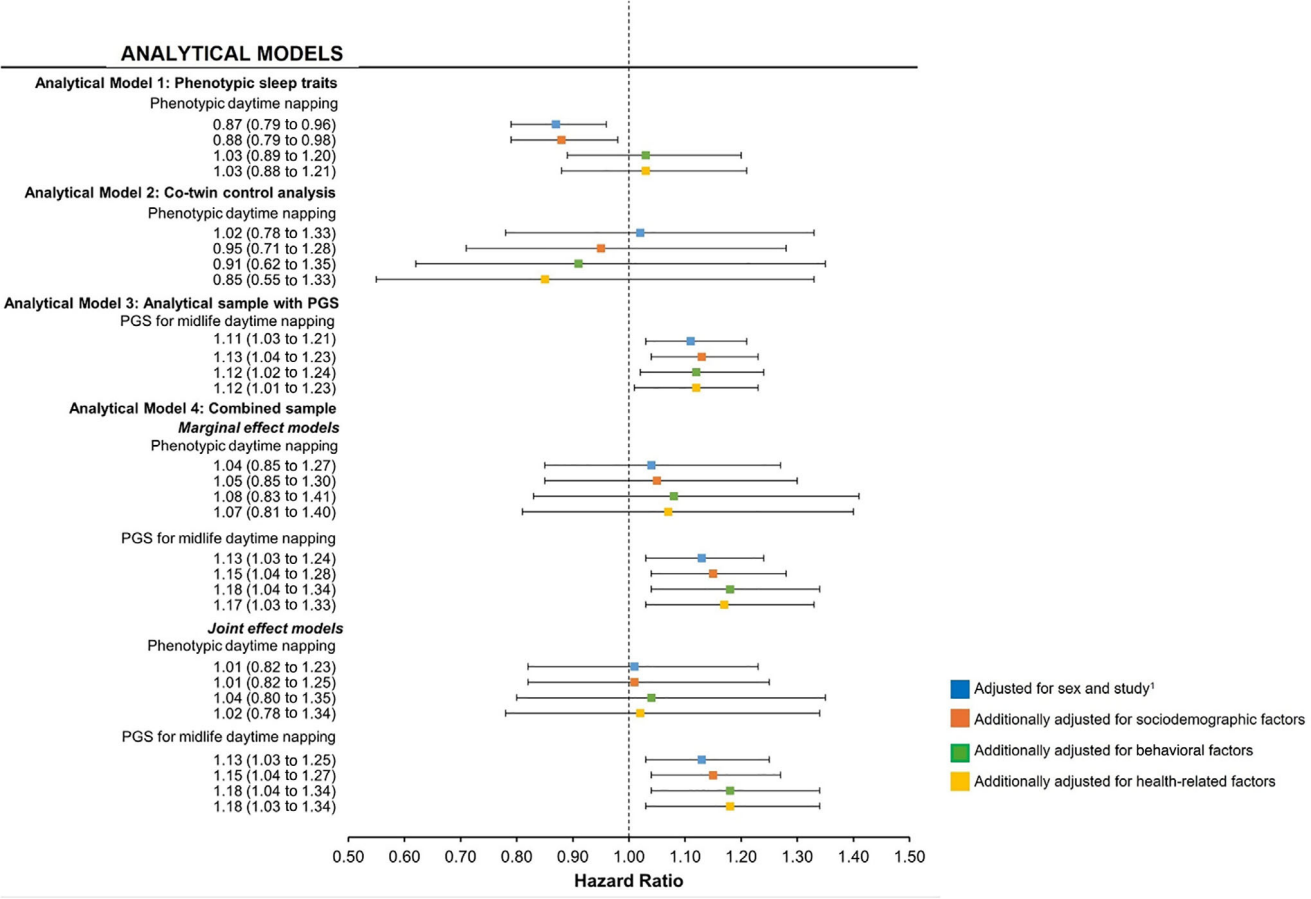
Forest plot of phenotypic and PGS associations for daytime napping across model adjustments and analytical frameworks after excluding dementia diagnosis within the first 10 years of follow‐up. Hazard ratios (HRs) are derived from Cox regression survival. Dotted vertical line: reference (HR = 1); horizontal black lines with caps: confidence intervals; square box: HR estimate; blue square box: model adjusted for sex and study; orange square box: model additionally adjusted for sociodemographic factors; green square box: model additionally adjusted for behavioral factors; yellow square box: model additionally adjusted for health‐related factors. ^1^Study is additionally adjusted when the model includes PGS. PGS, polygenic score.

#### 3.3.3 Interaction between daytime sleepiness and its corresponding PGS

No interaction was observed for daytime napping in the primary (*p* for interaction in sex‐ and study‐adjusted model = 0.54, eTable 14) or unrestricted analyses (*p *= 0.36, eTable 15). A statistically significant interaction was observed between phenotypic daytime sleepiness and the corresponding PGS in primary (HR for sex‐adjusted model 1.23; 95% CI: 1.00 to 1.52; *p* = 0.05) and unrestricted (HR for sex‐adjusted model 1.26; 95% CI: 1.05 to 1.51; *p* = 0.01) analyses, but these findings should be interpreted with caution given the absence of associations in the main‐effect models, the limited sample size, and the wide confidence intervals.

### 3.4 Post hoc analysis

Post hoc analyses were conducted for the PGS for daytime napping, stratified by *APOE* ε4 carrier status, across all analytical frameworks, given its consistent associations (eTable 16). The association between PGS for daytime napping and dementia was stronger among *APOE* ε4 non‐carriers than among carriers in the total genotyped data. In the combined phenotypic and genotyped subsample, joint and marginal effect sizes were comparable in non‐carriers and carriers of *APOE* ε4, especially in the restricted sample.

### 3.5 Sensitivity analyses

Results from the Fine‐Gray competing risk analyses were broadly consistent with those from the primary Cox proportional hazards models (eTables 17 to 20). Associations for the daytime napping PGS and dementia remained largely unchanged after accounting for death as a competing event. Associations for phenotypic daytime napping and phenotypic sleep duration were slightly attenuated but with comparable estimates, although for daytime napping the association no longer reach statistical significance. Overall, these findings suggest that competing mortality did not materially alter the observed associations.

## 4 DISCUSSION

In this population‐based study of Swedish twins, we prospectively examined associations of phenotypic and genetically predicted sleep traits, as well as their interactions, with dementia risk. When accounting for reverse causation, longer sleep duration and phenotypic daytime napping were associated with a lower dementia risk over a mean follow‐up of 13 years. Both associations were attenuated in within‐twin pair analyses, suggesting confounding by shared genetic or familial factors. The association with daytime napping, but not that with sleep duration, was also attenuated when adjusting for behavioral and health‐related factors. PGS for midlife daytime napping was associated with higher dementia risk over a 16‐year mean follow‐up, independent of sociodemographic, behavioral, and health‐related factors.

In the subsample with both phenotypic and genotypic data, no phenotypic sleep traits were associated with dementia risk, though the estimates for sleep duration were comparable with those observed in the restricted full analytical sample. However, a higher genetic predisposition to midlife daytime napping remained consistently associated with elevated dementia risk, both in the marginal‐effect model and after adjustment for phenotypic daytime napping in the joint‐effect model. No evidence of interaction between phenotypic and genetically predicted daytime napping was observed. A potential interaction was found for daytime sleepiness, although the absence of associations in the marginal‐effect models and wide confidence intervals limit the certainty of this finding. Detailed discussion of findings related to sleep duration is presented in eDiscussion in Supplement 1; here, we focus on findings for daytime napping.

The opposing directions of associations observed for phenotypic and genetically predicted daytime napping are noteworthy. In the subsample with both phenotypic and genotypic data, no inverse association was observed with phenotypic daytime napping, likely due to the younger age of this group, which resulted in fewer dementia diagnoses and reduced statistical power. Differences in age composition between the GWAS discovery cohort (typically middle‐aged adults) and our study population (mainly older adults) may partly explain this divergence, as daytime sleep habits such as napping change with aging. Older adults nap more often than younger‐ and middle‐aged adults, even when healthy.^29^, ^30^ Another important age‐related change is increased fragmentation of nighttime sleep duration, characterized by repeated and prolonged periods of wakefulness.^29^ This pattern is considered part of normal aging and may reflect greater sensitivity to light and environmental disturbances,^29^ as well as age‐related changes in gonadotropins and sex steroids.^30^

Unlike phenotypic traits, PGSs are inherited and remain stable throughout life; thus, genetic liability for daytime napping in midlife may capture a distinct vulnerability relevant to dementia, whereas napping in older age may serve as a compensatory or restorative function depending on its duration and context. It is also important to note that the relationship between napping and dementia likely varies according to nap frequency, duration, timing, and whether the nap is intentional. A recent study using objectively measured napping patterns reported that more frequent morning naps (9 to 11 a.m.) were linked to a higher risk of AD, whereas early afternoon naps (1 to 3 p.m.) and consistent nap patterns were associated with lower levels of AD pathology.^15^ Our study was unable to distinguish these features, given that no GWAS to date has identified genetic instruments that capture specific nap characteristics such as duration, timing, or frequency. Mendelian randomization evidence linking daytime napping to dementia risk is inconsistent. Anderson and colleagues^31^ reported suggestive protective associations with AD, while Ran and colleagues^32^ found increasing risk with excessive napping. These differences likely reflect heterogeneity in phenotype definitions and genetic instruments, highlighting the need for careful phenotype harmonization.

Over 123 genetic loci have been identified as associated with daytime napping, with many of these variants exhibiting pleiotropic effects, that is, influencing multiple traits, including associations with other sleep‐related traits and accelerometer‐derived sleep measures.^10^ However, 26 of the 123 loci (at the GWAS‐significant level) appear unique to daytime napping, suggesting that distinct biological mechanisms may underlie this sleep trait.^10^

Importantly, many napping‐associated genetic variants show pleiotropic associations with cardiometabolic traits, including higher blood pressure, BMI, and type 2 diabetes,^10^ which are established risk factors for dementia.^33^ Several of the identified lead variants for daytime napping fall within or near genes (e.g., *HCRTR1, HCRTR2, TRPC6*) involved in the hypocretin/orexin signaling pathway and broader arousal regulation, components of the sleep‐wake system increasingly implicated in AD.^34^, ^35^, ^36^ These shared genetic influences likely reflect pleiotropic effects, which may confound the direct association between napping and dementia. As such, genetic liability to napping may serve as a proxy for broader biological vulnerabilities rather than a causal mechanism, underscoring the importance of distinguishing between phenotypic trait and underlying genetic predisposition in sleep‐dementia research.

Several limitations should be acknowledged. First, all measures of sleep traits were self‐reported, which may be prone to recall bias and misclassification. Second, due to legal and data‐sharing restrictions, we were unable to access summary statistics from the largest GWAS that included 23andMe participants. This might reduce the genetic coverage and predictive power of our PGSs for certain sleep traits, such as chronotype.^8^ Third, discrepancies in the operationalization of sleep traits between the GWAS discovery dataset (e.g., “snoring” in UK Biobank) and our sample (e.g., “heavy snoring” in SALT) may affect comparability and the accuracy of the PGS construction. Fourth, while we captured the multidimensional nature of sleep using an additive sleep health score, alternative approaches such as principal component‐based scores^13^ could not be applied, as the components derived from the GWAS discovery dataset did not align with those from our sample. This approach may not fully reflect the potentially different biological relevance or contribution of individual sleep dimensions to dementia risk. Fifth, zygosity was not considered in this analysis because the sample size was insufficient to examine monozygotic and dizygotic twin pairs separately. Also, although our subsample with both phenotypic and genotypic data provided valuable insights, co‐twin control analyses with PGS in dizygotic twin pairs were not feasible due to limited sample size, restricting our ability to disentangle genetic and environmental contributions in detail. Sixth, no GWAS to date has identified genetic instruments that capture specific nap characteristics such as duration, timing, or frequency. Consequently, our analyses relied on a binary measure of daytime napping (yes/no), without further granularity on nap characteristics. Seventh, although a statistically significant interaction was observed between phenotypic daytime sleepiness and the corresponding PGS, these findings should be interpreted with caution. No significant main effects were observed for either phenotypic daytime sleepiness or daytime sleepiness PGS, and the relatively limited sample size and wide confidence intervals further limit the robustness of the interaction findings. Therefore, replication in larger, independent cohorts is needed before firm conclusions can be drawn.

This study has several notable strengths. First, it examined both phenotypic and genetically predicted sleep traits within a population‐based cohort, using core sleep measures alongside an additive PGS and assessing their interactions within a single analytic framework. The use of the STR provided several important advantages, including the ability to conduct co‐twin control analyses for genetic and familial confounding, long‐term follow‐up, genotype data, and linkage to nationwide registers. Importantly, all participants underwent structured baseline evaluation in SALT, allowing for confident exclusion of prevalent dementia. While nationwide registers may have lower sensitivity for detecting early or milder dementia cases, the comprehensive clinical work‐up at baseline substantially reduces the risk of misclassifying participants with undiagnosed dementia at study entry, thereby enhancing the robustness of our results.^22^, ^24^ Although phenotypic sleep traits were based on self‐reported questionnaire items, we used items from the KSQ, a validated and widely used sleep assessment tool.^21^ Complete follow‐up for mortality enabled the assessment for competing risks and did not indicate survival bias. Finally, adjusting for an extensive set of potential confounders further enhanced the robustness of our analytical models.

In conclusion, longer phenotypic sleep duration was associated with a lower risk of dementia, an association that was attributable to shared genetic and familial factors in the co‐twin control analyses. Phenotypic daytime napping was associated with a lower risk of dementia, while higher genetic predisposition to daytime napping was associated with greater risk of dementia, with estimates remaining stable across sociodemographic, lifestyle, and health‐related adjustments. The opposite direction of associations likely mirrors age‐specific associations, where daytime napping in late life may reflect a healthy part of the aging process. In contrast, the association between the PGS, representing daytime napping in midlife, and higher risk of dementia, which was independent of observed phenotypic daytime napping, may suggest that genetic liability to daytime napping reflects underlying biological vulnerability to dementia rather than current napping habits.

## CONFLICT OF INTEREST STATEMENT

The authors declare no conflicts of interest. Author disclosures are available in the Supporting Information.

## CONSENT STATEMENT

All human subjects provided informed consent or confirmed that consent was not necessary.

## Supporting information




Supporting Information

Supporting Information
