# Supplementary material for: Phenotypic and genetically predicted sleep traits in relation to dementia incidence in Swedish twins
Source: Alzheimers Dement. 2026 Nov 9;22(11):e71977. doi: 10.1002/alz.71977 (PMC13746180; doi:10.1002/alz.71977)
Supplement: Supplementary file 2 [file ALZ-22-e71977-s001.docx]

**Supplemental Online Content**

[**eTable 1**. Summary of UK Biobank GWAS Sources for sleep traits and polygenic score construction	6](#_Toc241605651)

[**eTable 2.** Association of polygenic score (PGS) for sleep traits with its corresponding phenotypic sleep trait	8](#_Toc241605652)

[**eTable 3.** Information on self-reported sleep traits	9](#_Toc241605653)

[**eTable 4.** ICD codes used to identify dementia diagnoses	11](#_Toc241605654)

[**eTable 5.** ATC-codes for identification of dementia medication.	11](#_Toc241605655)

[**eTable 6.** Summary of covariates violating the proportionality hazards assumption and corresponding stratification across models	12](#_Toc241605656)

[**eTable 7.** Characteristics of the study population by phenotypic sample, PGS sample, and sample with both phenotypic and PGS data.	13](#_Toc241605657)

[**eTable 8.** Number of incident dementia and total N for each model adjustment	14](#_Toc241605658)

[**eTable 9.** Association of phenotypic sleep traits with incident dementia within complete twin pairs in the total sample with available self-reported sleep traits (co-twin control analysis) excluding dementia cases occurring within the first 10 years of follow-up.	16](#_Toc241605659)

[**eTable 10.** Association of phenotypic sleep traits with incident dementia in the total sample with available self-reported sleep traits	17](#_Toc241605660)

[**eTable 11.** Association of phenotypic sleep traits with incident dementia within complete twin pairs in the total sample with available self-reported sleep traits (co-twin control analysis)	18](#_Toc241605661)

[**eTable 12.** Association of PGS for midlife sleep traits and dementia risk in the genotyped sample	19](#_Toc241605662)

[**eTable 13.** Marginal and joint effect associations of phenotypic sleep traits and their corresponding PGS with dementia risk in subsample with both phenotypic and genotyped data available.	20](#_Toc241605663)

[**eTable 14**. Main and interactive effect terms of phenotypic sleep traits and their corresponding PGS with dementia risk excluding dementia cases occurring within the first 10 years of follow-up.	22](#_Toc241605664)

[**eTable 15.** Main and interactive effect terms of phenotypic sleep traits and their corresponding PGS with dementia risk	24](#_Toc241605665)

[**eTable 16.** Post hoc sensitivity analyses of the PGS for midlife daytime napping stratified by APOE ε4 allele carrier status	26](#_Toc241605666)

[**eTable 17.** Association of phenotypic sleep traits with incident dementia in the total sample with available self-reported sleep traits excluding dementia cases occurring within the first 10 years of follow-up using competing-risk analyses	27](#_Toc241605667)

[**eTable 18.** Association of PGS for midlife sleep traits and dementia risk in the genotyped sample excluding dementia cases occurring within the first 10 years of follow-up using competing-risk analyses	28](#_Toc241605668)

[**eTable 19.** Marginal and joint effect associations of phenotypic sleep traits and their corresponding midlife PGS with dementia risk excluding dementia cases occurring within the first 10 years of follow-up using competing-risk analyses.	29](#_Toc241605669)

[**eTable 20.** Main and interactive effect terms of phenotypic sleep traits and their corresponding PGS with dementia risk excluding dementia cases occurring within the first 10 years of follow-up using competing-risk analyses.	31](#_Toc241605670)

[eMethods	2](#_Toc226494099)

[Construction of polygenic scores (PGS)	2](#_Toc226494100)

[Phenotypic Sleep Trait Assessment	2](#_Toc226494101)

[Dementia case ascertainment	3](#_Toc226494102)

[Covariates	3](#_Toc226494103)

[Statistical analyses	4](#_Toc226494104)

[eDiscussion	26](#_Toc226494105)

[eReferences	27](#_Toc226494106)

# eMethods

## Construction of polygenic scores (PGS)

Standard GWAS quality control following established guidelines was performed.^1^ We selected HapMap3 SNPs present on the two genotyping arrays with a minor allele frequency >1% and imputation quality (“info score”) >0.8. Additionally, all ambiguous SNPs were removed to avoid introduction of a potential source of systematic error.^1^ All summary statistics from GWAS were cleaned using RStudio (v4.3.2; <https://www.r-project.org/>) with the following R packages: data.table (1.15.4), tidyverse (2.0.0), and utils (4.3.2).

To deal with linkage disequilibrium, effect size shrinkage with SBayesR^2^ (2.03beta) was performed. PGS were then computed with Plink 2.0 (2.0.0-a.6.12) by summing up the number of risk alleles at each genetic loci and weighing them by their effect size in the GWAS of the sleep trait of interest. Prior to analyses, all sleep PGSs were adjusted for genetic ancestry by regressing out the first five principal components from the score and standardized (mean = 0, SD = 1) within genotyping data collection (TwinGene, SALT-Y).

We assessed the extent to which each PGS predicted its corresponding phenotypic sleep trait using regression models adjusted for age, sex, and genotyping data collection (TwinGene or SALT-Y). All sleep-related PGS, except the PGS for daytime sleepiness, showed a statistically significant association with their respective phenotypes (**eTable 2**). The PGS for daytime sleepiness was a poor predictor of phenotypic daytime sleepiness, explaining very little variance (*R^2^* close to zero). For the other traits, the proportion of variance explained by the PGS, measured by *R^2^* for continuous traits and Nagelkerke’s Pseudo- *R^2^* for binary traits, ranged from 1.6% for sleep health score to 10.6% for sleep duration. These values indicate modest but meaningful genetic contributions to individual differences in most sleep traits.

## Phenotypic Sleep Trait Assessment

A subsample of SALT participants completed a sleep survey using the Karolinska Sleep Questionnaire (KSQ), a validated instrument for assessing subjective sleep and sleepiness.^3^ To harmonize phenotypic sleep trait definitions with those used in the UK Biobank, the KSQ measures were converted to UK Biobank-compatible scales. Bedtime and wake-up time were recorded as integer values based on participants’ self-reported usual sleep and wake times. Nighttime sleep duration was calculated as the difference between self-reported bedtime and wake-up time. Daytime napping was assessed using the question “Do you usually take a nap at least every second day?” with binary response options (Yes/No).

Other sleep items included asking participants whether in the past 6 months they have experienced any of the following symptoms: heavy snoring, sleepy during the daytime (daytime sleepiness), difficulty falling asleep (characteristic of sleep onset-insomnia), and continuously waking up and difficulty falling asleep again (maintenance insomnia). These items were rated on a five-point scale ranging from 0 (never) to 4 (always). Snoring was assessed via a KSQ item on heavy snoring and was dichotomized into “never snorer” (0) and “ever snorer” (1-4). Daytime sleepiness was assessed using the item on feeling sleeping during the day. Responses were re-coded to a four-level ordinal scale: never/rarely (0/1), sometime (2), often (3) and always (4), with higher values indicating greater severity.

Insomnia symptoms were derived from two KSQ items reflecting sleep-onset and sleep maintenance difficulties. Responses from these two items were binarized, averaged (yielding values of 0, 0.5, or 1), and re-binarized to classify participants as having no/infrequent insomnia (0-0.5) or frequent insomnia (1). Chronotype was assessed using a single KSQ item on morningness-eveningness preference, rated on a five-level ordinal from “Definitely a morning person” to “Definitely a night person”. Responses were recorded on a scale ranging from -2 (definitely evening type) to +2 (definitely morning type), consistent with the UK Biobank chronotype measure.

More details on KSQ items, response options, and coding procedures are provided in **eTable 3**. All items also included response options such as “don’t know” or “refused to answer”, which were coded as missing unless otherwise specified. All adaptations were made to align with the phenotypic definitions used in multiple GWAS conducted in UK Biobank.

*Sleep health score*

An additive sleep health score was calculated for participants with available data on five core sleep traits: nighttime sleep duration, chronotype, insomnia symptoms, snoring, and daytime sleepiness. Each sleep trait was dichotomized, with one point assigned for the presence of a positive trait and zero otherwise. Positive sleep traits were defined as: sleep duration of 7 to 8 hours (inclusive), infrequent insomnia symptoms, morning chronotype preference, absence of snoring, and infrequent daytime sleepiness. The final sleep health score represents the total number of positive sleep traits per individual, ranging from 0 to 5, with higher scores indicating better overall sleep health.

## Dementia case ascertainment

Dementia cases were ascertained by combining information from the following four registers:

1. The National Patient Register (NPR) refers to the inpatient register recording 99% of all overnight hospitalizations. Main and secondary diagnosis are recorded in *International Classification of Diseases* (ICD) codes. It was established in 1964 and reached nationwide coverage from 1987 and the current data included linkage throughout 2016.
2. The Outpatient Register (OPR) is part of NPR and records outpatient specialist care in the same manner. The OPR was started in 2001 and the current data included linkage throughout 2016.
3. The Cause of Death Register (CDR) records the underlying and contributory causes of given in ICD codes. The register reached nationwide coverage in 1961, and the current data included linkage throughout 2016. It includes all individuals, who were registered as residents in Sweden in the year of their death.
4. The Prescribed Drug Register (PDR) which records all dispensed prescriptions according to ATC codes. The register reached nation-wide coverage in 2005, and the current data included linkage throughout 2017.

The sensitivity and specificity of dementia case identification when NPR/OPR and CDR are combined is 63% and 99%, respectively.^4^ Unfortunately, no validation study of dementia information from PDR is available to date. The ICD codes used to identify dementia diagnosis are listed in **eTable 4.** ATC codes to identify dementia medication are listed in **eTable 5**.

## Covariates

Covariates were assessed using questionnaire at 1998-2002 assessment, as well as from registry records including the National Patient Register/ Outpatient Register, Cause of Death Register, and the Prescribed Drug Register. Sociodemographic variables included education (≤7 years, versus >7 years, corresponding to basic versus more than basic education at the time), occupational status (white collar, blue collar, and self-employed), and marital status (married/cohabitating, divorced/separated/widowed/single). Behavioral factors included alcohol risk consumption (defined as >14 units of alcohol per week for men and >9 units per week for women), physical activity (assessed using the question “Which of the options fits your annual exercise pattern?” and categorized as low [almost never exercise or much less exercise than average], moderate [less or average amount of exercise], and high [more or much more exercise than average]), and smoking status (non-smoker/party smoker, current/former smoker). Health-related factors consisted of current depressive symptomatology defined by the 11-item short-form item of Center for Epidemiologic Studies Depression scale (CES-D) score, use of hypnotics and sedatives, body mass index, prevalent diabetes (self-reported) and prevalent cardiovascular diseases including coronary heart disease, and stroke identified using linkage to registries.

Body mass index was calculated from self-reported weight and height. Previous investigations with the STR have shown self-reported height and weight to be highly consistent with the corresponding measured values (correlation coefficients 0.97 and 0.95, respectively).^5^ From this information body mass index was calculated as weight (kg) divided by the square of height (m^2^).

Participants were asked four questions about medications. Responses were manually coded according to the ATC classification valid at the time of the questionnaire assessment. Use of hypnotics and sedatives was defined as responses corresponding to ATC code N05C.

## Statistical analyses

Baseline characteristics of the study participants by dementia status were assessed using student’s *t* test for continuous variables and *χ2* test for categorical variables.

*Proportionality Assumption*

For all analyses, we evaluated the proportional hazards assumption using the global and covariate-specific Grambsch-Therneau tests (stphtest command in Stata). These formal tests were complemented by visual inspection of scaled Schoenfeld residual plots. The assessments indicated non-proportionality for certain adjusting covariates. To account for differences in baseline hazards associated with these variables, the relevant categorical covariates were included in the strata statement (stratified Cox model) for the specific model that was violated. Among the covariates assessed, marital status, smoking status, and alcohol consumption most consistently violated the proportionality hazards assumption. **eTable 6** presents the models and the covariates for which stratification was applied.

**eTable 1**. Summary of UK Biobank GWAS Sources for sleep traits and polygenic score construction

| **Sleep trait** | **Question from UK Biobank** | **Individuals from UK Biobank** | **Number of independent loci**  **(p<5 * 10-8 )** | **Resulting SNPs after quality control** |
| --- | --- | --- | --- | --- |
| Overall sleep duration | About how many hours sleep do you get in every 24 hours? (please include naps)^6^ | 446,118 | 78 | 1,178,712 |
| Snoring | "Does your partner or a close relative or friend complain about your snoring?"^7^ | 408,317 | 42 | 1,179,823 |
| Daytime dozing/sleeping | "How likely are you to doze off or fall asleep during the daytime when you don't mean to? (e.g. when working, reading or driving)"^8^ | 452,071 | 42 | 1,178,708 |
| Overall chronotype | “Do you consider yourself to be?” with one of six possible answers: “Definitely a ‘morning’ person”, “More a ‘morning’ than ‘evening’ person”, “More an ‘evening’ than a ‘morning’ person”, “Definitely an ‘evening’ person”, “Do not know” or “Prefer not to answer”.^9^ | 449,734 | 351 (using genome-wide data from 697,828 UK Biobank and 23andMe participants) | 1,175,833 |
| Insomnia | Do you have trouble falling asleep at night, or do you wake up in the middle of the night?” and asked them to select from responses of “never/rarely,” “sometimes,” “usually,” and “prefer not to answer.”^10^ | 453,379 | 57 | 1,178,712 |
| Additive sleep health score | Combination of the above five core sleep traits.^11^ Computation described in sleep trait assessment section. | 413,904 | 45 | 1,178,473 |
| Daytime napping | Do you have a nap during the day? With responses Never/rarely, Sometimes, Usually, prefer not to answer.^12^ | 452,633 | 123 | 1,178,497 |

Abbreviations: SNP, single nucleotide polymorphisms.

**eTable 2.** Association of polygenic score (PGS) for sleep traits with its corresponding phenotypic sleep trait

| **Phenotypic sleep trait** | **β (95% CI)** | **P value** | ***R^2^*** | **Nagelkerke’s Pseudo-*R^2^*** |
| --- | --- | --- | --- | --- |
| Sleep duration | 0.064 (0.028 - 0.100) | <0.001 | 0.106 |  |
| Chronotype | 0.359 (0.312 - 0.406) | <0.001 | 0.064 |  |
| Daytime sleepiness | 0.027 (-0.003 - 0.056) | 0.078 | 0.002 |  |
| Sleep health score | 0.106 (0.049 - 0.163) | <0.001 | 0.016 |  |
|  |  |  |  |  |
|  |  |  |  |  |
|  | **OR (95% CI)** | **P value** |  |  |
| Insomnia | 1.159 (1.036 - 1.295) | 0.010 |  | 0.049 |
| Snore | 1.356 (1.224 - 1.503) | <0.001 |  | 0.084 |
| Nap | 1.296 (1.201 - 1.398) | <0.001 |  | 0.071 |

All models adjusted were adjusted for age, sex, and study. *R^2^* provided for linear models and Nagelkerke’s Pseudo-*R^2^* provided for logistic models. To account for relatedness among twins, we applied the robust variance errors estimator for clustered observations.

**eTable 3.** Information on self-reported sleep traits

| **Sleep trait** | **Questionnaire** | **Response options** | **Coding procedure (if any)** |
| --- | --- | --- | --- |
| **Nighttime sleep duration** | What time do you usually get up? [Wake-up time]  What time do you usually go to bed? [Bedtime] | Enter time: in hours | Nighttime sleep duration was calculated as the difference between bedtime and wake-up time. This is essentially “time in bed” rather than “time slept”. |
| **Daytime napping** | Do you usually take a nap at least every second day? | No, yes | Binary response categories were retained. |
| **Chronotype** | Try to determine to what degree you are a “morning person" or a "night person". Are you… | **0** Definitely a morning person (energetic in the morning and tired at night)  **1** To some degree a morning person  **2** To some degree a night person  **3** Definitely a night person (tired in the morning and energetic at night)  **998** don’t know | The KSQ response scale was adapted to align with the interpretation of the UK Biobank subjective chronotype measure.  **-2** definitely night (3)  **-1** some degree night (2)  **0** don’t know (998)  **1** some degree morning (1)  **2** definitely morning (0)  On a scale from -2 to +2, higher values reflect a stronger morning preference, which is considered a favorable trait. |
| How often have you experienced the following symptoms during the last 6 months? | | | |
| **Snoring** | Heavy snoring | 0 Never  1 Seldom  2 Sometimes  3 Usually  4 Always | Responses were dichotomized into binary categories to align with UKB:   1. No: never snorers (**Never**) 2. Yes: ever snorer (S**eldom/Sometimes/Usually/Always**) |
| **Daytime sleepiness** | Sleepy at daytime | 0 Never  1 Seldom  2 Sometimes  3 Usually  4 Always | Never/rarely – **(Never/Seldom)**  Sometimes – **(Sometimes)**  Often – **(Usually)**  All the time – (**Always)**  Higher values indicate increased severity, aligned with the UKB’s continuous ordinal scale interpretation. |
| **Insomnia** | Difficulty falling asleep  Continuously waking up and having difficulty falling asleep again | 0 Never  1 Seldom  2 Sometimes  3 Usually  4 Always | **Step 1:** Each item was binarized separately with higher value indicating presence of insomnia.  **Step 2:** A mean score was calculated across the two items, resulting in values of 0, 0.5, or 1.  **Step 3:** The mean score was binarized again, classifying participants as having no/infrequent insomnia symptoms (0-0.5) or frequent insomnia (1). |

**eTable 4.** ICD codes used to identify dementia diagnoses

|  | **ICD-7** | **ICD-8** | **ICD-9** | **ICD-10** |
| --- | --- | --- | --- | --- |
| **Dementia** |  |  |  |  |
| **Alzheimer´s disease** | 304 | 290 | 290A | F00 |
|  | 305 |  | 290B | G30 |
|  |  |  | 331A |  |
|  |  |  |  |  |
| **Vascular dementia** |  | 293.0 | 290E | F01 |
|  |  | 293.1 |  |  |
|  |  |  |  |  |
| **Other dementia** | 306 |  | 290X | F02 |
|  |  |  | 290W | F03 |
|  |  |  | 294B | G311 |
|  |  |  | 331B | G318A |
|  |  |  | 331C | F051 |
|  |  |  | 331X |  |

**eTable 5.** ATC-codes for identification of dementia medication.

**N06DA** Anticholinesterases

N06DA02 Donepezil

N06DA03 Rivastigmine

N06DA04 Galantamine

(N06DA01 Tacrine and N06DA05 Ipidacrine not prescribed in Sweden)

**N06DX** Other anti-dementia drugs

N06DX01 Memantine

(N06DX02 Ginkgo folium not prescribed in Sweden)

**eTable 6.** Summary of covariates violating the proportionality hazards assumption and corresponding stratification across models

|  | **Overall** | **Sleep trait-Specific** |
| --- | --- | --- |
|  |  |  |
| **Phenotypic sample** |  |  |
| Unrestricted sample | Marital status, education | - |
| Restricted sample |  | Marital status and education for snoring; education for additive sleep health score |
|  |  |  |
| **Genotyped sample** |  |  |
| Unrestricted sample | Smoking | - |
| Restricted sample | Prevalent CVD | - |
|  |  |  |
| **Combined phenotypic and genotyped data** | | |
| Unrestricted sample | - | - |
| Restricted sample |  |  |
| *Marginal effect models* |  |  |
| Phenotypic | Alcohol consumption | Additionally marital status for snoring; smoking for additive sleep health score. |
| PGS | Alcohol consumption | Additionally study for daytime sleepiness; study and smoke for additive sleep health score; study, marital status and smoke for snoring. |
| *Joint effect models* | Alcohol consumption | Additionally smoking for additive sleep health score; smoke and marital status for snoring. |

**eTable 7.** Characteristics of the study population by phenotypic sample, PGS sample, and sample with both phenotypic and PGS data.

|  | **PHENOTYPIC SAMPLE** | | | **PGS SAMPLE** | | | **COMBINED** | | |
| --- | --- | --- | --- | --- | --- | --- | --- | --- | --- |
|  | **Incident dementia** | | | **Incident dementia** | | | **Incident dementia** | | |
|  | **No** | **Yes** | **P-value** | **No** | **Yes** | **P-value** | **No** | **Yes** | **P-value** |
| Baseline age (years), M (SD) | 70.12 (7.74) | 73.03 (6.32) | **<0.0001** | 54.93 (7.69) | 66.65 (7.13) | **<0.0001** | 66.40 (6.36) | 70.02 (5.29) | **<0.0001** |
| Female | 6203 (53.72) | 1581 (60.97) | **2.02e-11** | 8204 (52.57) | 338 (49.06) | 0.071 | 1352 (49.06) | 243 (51.05) | 0.422 |
| Education ≥8 years | 6156 (53.53) | 1258 (48.74) | **<0.001** | 13399 (86.06) | 457 (67.01) | **<0.001** | 1793 (65.13) | 308 (64.71) | 0.858 |
| Blue-collar jobs | 5318 (50.29) | 1201 (51.83) | 0.185 | 5818 (38.29) | 269 (41.51) | **0.003** | 1075 (41.25) | 178 (39.82) | 0.776 |
| Night work | 3082 (27.35) | 692 (27.33) | 0.984 | 4706 (30.48) | 222 (32.99) | 0.167 | 768 (28.26) | 143 (30.56) | 0.309 |
| Married/Cohabitating | 7422 (64.65) | 1596 (61.76) | **0.006** | 12357 (80.33) | 519 (76.32) | 0.010 | 2078 (75.87) | 353 (74.47) | 0.514 |
| Current/ex-smoker | 4804 (42.26) | 915 (35.6) | **5.95e-10** | 7486 (49.54) | 289 (42.81) | **0.001** | 1130 (41.47) | 181 (38.27) | 0.191 |
| Alcohol risk consumption | 167 (1.72) | 28 (1.33) | 0.197 | 380 (2.55) | 13 (2.11) | 0.498 | 41 (1.68) | 6 (1.42) | 0.695 |
| High physical activity | 4253 (55.02) | 770 (57.46) | 0.243 | 7698 (50.77) | 323 (57.78) | **0.001** | 1385 (58.07) | 222 (62.89) | 0.139 |
| Current depressive symptomatology | 3703 (34.46) | 882 (36.84) | **0.027** | 4434 (29.53) | 198 (30.41) | 0.628 | 769 (29.65) | 139 (30.48) | 0.718 |
| Use of hypnotics & sedatives | 547 (4.74) | 143 (5.51) | 0.097 | 314 (2.01) | 26 (3.77) | **0.002** | 79 (2.87) | 22 (4.62) | **0.042** |
| BMI, M (SD) | 25.16 (3.7) | 25.01 (3.48) | 0.073 | 24.92 (3.38) | 25.17 (3.24) | 0.062 | 25.21 (3.33) | 25.10 (3.22) | 0.522 |
| Diabetes | 1026 (8.89) | 234 (9.03) | 0.825 | 460 (2.95) | 51 (7.47) | **3.60e-11** | 150 (5.44) | 33 (6.93) | 0.195 |
| Prevalent CVD | 1884 (16.32) | 450 (17.35) | 0.199 | 736 (4.72) | 89 (12.92) | **7.36e-22** | 295 (10.7) | 72 (15.13) | **0.005** |
|  |  |  |  |  |  |  |  |  |  |
| *Phenotypic sleep traits* |  |  |  |  |  |  |  |  |  |
| Sleep duration, in hours, M (SD) | 8.44 (1.15) | 8.61 (1.16) | **<0.0001** |  |  |  | 8.25 (1.05) | 8.42 (1.09) | **0.001** |
| Chronotype, M (SD) | 0.42 (1.45) | 0.48 (1.43) | 0.101 |  |  |  | 0.41 (1.44) | 0.44 (1.45) | 0.639 |
| Insomnia symptomology | 1376 (20.83) | 289 (20.94) | 0.923 |  |  |  | 293 (18.35) | 49 (21.68) | 0.229 |
| Heavy snoring | 2821 (47.76) | 536 (43.9) | **0.014** |  |  |  | 788 (53.21) | 109 (51.66) | 0.673 |
| Daytime sleepiness, M (SD) | 1.35 (.65) | 1.34 (.65) | 0.451 |  |  |  | 1.32 (0.60) | 1.34 (0.59) | 0.720 |
| Daytime napping | 4931 (42.78) | 1140 (44.07) | 0.232 |  |  |  | 1047 (38.06) | 217 (45.59) | **0.002** |
| Sleep health score, M (SD) | 3.07 (1.1) | 3.04 (1.08) | 0.421 |  |  |  | 3.09 (1.11) | 3.01 (1.19) | 0.350 |
|  |  |  |  |  |  |  |  |  |  |
| *Polygenic scores, M (SD)* |  |  |  |  |  |  |  |  |  |
| Sleep duration |  |  |  | -0.0004 (0.17) | 0.009 (0.17) | 0.152 | 0.002 (0.17) | 0.005 (0.16) | 0.740 |
| Chronotype |  |  |  | 0.0003 (0.29) | -0.007 (0.31) | 0.536 | -0.010 (0.30) | -0.005 (0.30) | 0.718 |
| Insomnia symptomology |  |  |  | 0.0003 (0.09) | -0.006 (0.10) | 0.107 | -0.004 (0.10) | -0.010 (0.10) | 0.228 |
| Snoring |  |  |  | 0.00004 (0.07) | -0.001 (0.07) | 0.725 | -0.002 (0.07) | -0.004 (0.07) | 0.631 |
| Daytime sleepiness |  |  |  | -0.0001 (0.06) | 0.0013 (0.06) | 0.576 | -0.001 (0.06) | 0.001 (0.06) | 0.474 |
| Daytime napping |  |  |  | -0.001 (0.11) | 0.0114 (0.11) | **0.005** | -0.001 (0.11) | 0.01 (0.11) | **0.018** |
| Sleep health score |  |  |  | -0.0001 (0.14) | 0.0034 (0.14) | 0.512 | 0.005 (0.14) | 0.01 (0.14) | 0.423 |

Data is N (col%), unless otherwise stated. **Bold** cells denote P<0.05.

**eTable 8.** Number of incident dementia and total N for each model adjustment

| **Association of phenotypic sleep traits with dementia risk** | | | | |
| --- | --- | --- | --- | --- |
|  | **Adjusted for sex** | **Additionally Adjusted for Sociodemographic Factors^1^** | **Additionally Adjusted for Behavioral Factors^2^** | **Additionally Adjusted for Health-Related Factors^3^** |
| Sleep duration | 2571/13961 | 2299/12644 | 1058/7284 | 962/6759 |
| Chronotype | 2590/14116 | 2314/12778 | 1064/7338 | 969/6805 |
| Insomnia | 1380/7987 | 1217/7200 | 481/3952 | 453/3743 |
| Snoring | 1221/7127 | 1082/6448 | 443/3623 | 419/3445 |
| Daytime sleepiness | 1390/8001 | 1224/7210 | 483/3953 | 454/3741 |
| Daytime napping | 2587/14114 | 2312/12777 | 1065/7340 | 969/6806 |
| Sleep health score | 1033/6106 | 914/5528 | 383/3187 | 366/3047 |
|  |  |  |  |  |
| **Association of phenotypic sleep traits with dementia risk excluding dementia cases within the first 10 years of follow-up** | | | | |
| Sleep duration | 1537/12927 | 1380/11738 | 740/6974 | 689/6493 |
| Chronotype | 1540/13066 | 1383/11860 | 742/7024 | 691/6534 |
| Insomnia | 843/7450 | 746/6734 | 354/3827 | 338/3629 |
| Snoring | 742/6648 | 661/6030 | 325/3507 | 311/338 |
| Daytime sleepiness | 846/7457 | 748/6739 | 355/3827 | 339/3627 |
| Daytime napping | 1538/13065 | 1382/11860 | 742/7025 | 691/6535 |
| Sleep health score | 646/5719 | 574/5191 | 286/3092 | 276/2958 |
|  |  |  |  |  |
| **Association of PGS for midlife sleep traits with dementia risk in the fully genotyped sample** | | | | |
|  | **Adjusted for sex and study** | **Additionally Adjusted for Sociodemographic Factors^1^** | **Additionally Adjusted for Behavioral Factors^2^** | **Additionally Adjusted for Health-Related Factors^3^** |
| Sleep duration | 689/16294 | 645/15637 | 483/14082 | 460/13498 |
| Chronotype | 689/16294 | 645/15637 | 483/14082 | 460/13498 |
| Insomnia | 689/16294 | 645/15637 | 483/14082 | 460/13498 |
| Snoring | 689/16294 | 645/15637 | 483/14082 | 460/13498 |
| Daytime sleepiness | 689/16294 | 645/15637 | 483/14082 | 460/13498 |
| Daytime napping | 689/16294 | 645/15637 | 483/14082 | 460/13498 |
| Sleep health score | 689/16294 | 645/15637 | 483/14082 | 460/13498 |
|  |  |  |  |  |
| **Association of PGS for midlife sleep traits with dementia risk in the fully genotyped sample excluding dementia cases within the first 10 years of follow-up** | | | | |
| Sleep duration | 559/16164 | 528/15520 | 397/14001 | 380/13423 |
| Chronotype | 559/16164 | 528/15520 | 397/14001 | 380/13423 |
| Insomnia | 559/16164 | 528/15520 | 397/14001 | 380/13423 |
| Snoring | 559/16164 | 528/15520 | 397/14001 | 380/13423 |
| Daytime sleepiness | 559/16164 | 528/15520 | 397/14001 | 380/13423 |
| Daytime napping | 559/16164 | 528/15520 | 397/14001 | 380/13423 |
| Sleep health score | 559/16164 | 528/15520 | 397/14001 | 380/13423 |
|  |  |  |  |  |
| **Combined sample with both phenotypic and genotypic data available** | | | | |
| Sleep duration | 476/3202 | 445/3007 | 294/2270 | 281/2137 |
| Chronotype | 476/3227 | 445/3028 | 294/2283 | 281/2147 |
| Insomnia | 226/1823 | 216/1710 | 135/1296 | 133/1239 |
| Snoring | 211/1692 | 202/1589 | 128/1210 | 127/1161 |
| Daytime sleepiness | 226/1824 | 216/1712 | 135/1297 | 133/1240 |
| Daytime napping | 476/3227 | 445/3028 | 294/2283 | 281/2146 |
| Sleep health score | 180/1510 | 172/1419 | 109/1085 | 109/1045 |

1Sociodemographic factors include education, occupational status, and marital status.

^2^Behavioral factors consist of alcohol risk consumption, physical activity, and smoking status.

^3^Health-related factors include current depressive symptomatology, use of hypnotics and sedatives, body mass index, prevalent diabetes, and prevalent cardiovascular diseases.

**eTable 9.** Association of phenotypic sleep traits with incident dementia within complete twin pairs in the total sample with available self-reported sleep traits (co-twin control analysis) excluding dementia cases occurring within the first 10 years of follow-up.

| **Phenotypic traits** | **Adjusted for sex** | | **Additionally Adjusted for Sociodemographic Factors^1^** | | **Additionally Adjusted for Behavioral Factors^2^** | | **Additionally Adjusted for Health-Related Factors^3^** | |
| --- | --- | --- | --- | --- | --- | --- | --- | --- |
|  | **HR (95% CI)** | **P** | **HR (95% CI)** | **P** | **HR (95% CI)** | **P** | **HR (95% CI)** | **P** |
| Sleep duration | 1.03 (0.90 - 1.19) | 0.63 | 1.06 (0.91 - 1.23) | 0.47 | 0.99 (0.81 - 1.21) | 0.90 | 1.09 (0.87 - 1.36) | 0.47 |
| Chronotype | 1.07 (0.97 - 1.17) | 0.18 | 1.06 (0.96 - 1.18) | 0.26 | 1.12 (0.97 - 1.28) | 0.12 | 1.07 (0.92 - 1.25) | 0.38 |
| Insomnia | **0.64 (0.42 - 0.97)** | **0.04** | 0.66 (0.41 - 1.06) | 0.09 | 0.77 (0.40 - 1.45) | 0.42 | 0.76 (0.38 - 1.54) | 0.45 |
| Snoring | 1.07 (0.71 - 1.61) | 0.74 | 1.06 (0.66 - 1.70) | 0.81 | 0.96 (0.48 - 1.93) | 0.92 | 1.07 (0.52 - 2.20) | 0.86 |
| Daytime sleepiness | 0.94 (0.72 - 1.22) | 0.64 | 0.90 (0.67 - 1.21) | 0.49 | 0.84 (0.56 - 1.26) | 0.40 | 0.84 (0.54 - 1.30) | 0.44 |
| Daytime napping | 1.02 (0.78 - 1.33) | 0.91 | 0.95 (0.71 - 1.28) | 0.74 | 0.91 (0.62 - 1.35) | 0.65 | 0.85 (0.55 - 1.33) | 0.48 |
| Sleep health score | 1.02 (0.83 - 1.25) | 0.86 | 1.01 (0.79 - 1.27) | 0.96 | 1.23 (0.89 - 1.68) | 0.21 | 1.14 (0.82 - 1.60) | 0.43 |

**Bold** cells denote P<0.05.

^1^Sociodemographic factors include education, occupational status, and marital status.

^2^Behavioral factors consist of alcohol risk consumption, physical activity, and smoking status.

^3^Health-related factors include current depressive symptomatology, use of hypnotics and sedatives, body mass index, prevalent diabetes, and prevalent cardiovascular diseases.

**eTable 10.** Association of phenotypic sleep traits with incident dementia in the total sample with available self-reported sleep traits

| **Phenotypic traits** | **Adjusted for sex** | | **Additionally Adjusted for Sociodemographic Factors^1^** | | **Additionally Adjusted for Behavioral Factors^2^** | | **Additionally Adjusted for Health-Related Factors^3^** | |
| --- | --- | --- | --- | --- | --- | --- | --- | --- |
|  | **HR (95% CI)** | **P** | **HR (95% CI)** | **P** | **HR (95% CI)** | **P** | **HR (95% CI)** | **P** |
| Sleep duration | 1.00 (0.96 - 1.04) | 0.95 | 1.00 (0.96 - 1.04) | 0.99 | 0.98 (0.92 - 1.04) | 0.45 | 0.98 (0.91 - 1.04) | 0.49 |
| Chronotype | 1.02 (0.99 - 1.05) | 0.15 | 1.01 (0.98 - 1.04) | 0.35 | 1.00 (0.96 - 1.05) | 0.87 | 1.00 (0.96 - 1.05) | 0.88 |
| Insomnia | **0.86 (0.76 - 0.98)** | **0.03** | 0.89 (0.78 - 1.03) | 0.12 | 0.90 (0.71 - 1.14) | 0.37 | 0.86 (0.67 - 1.11) | 0.26 |
| Snoring | 0.98 (0.88 - 1.10) | 0.77 | 0.96 (0.85 - 1.09) | 0.53 | 1.01 (0.83 - 1.22) | 0.92 | 1.07 (0.88 - 1.31) | 0.50 |
| Daytime sleepiness | 0.98 (0.90 - 1.07) | 0.73 | 1.00 (0.92 - 1.10) | 0.97 | 1.04 (0.90 - 1.20) | 0.60 | 1.05 (0.91 - 1.22) | 0.48 |
| Daytime napping | 0.97 (0.90 - 1.05) | 0.41 | 0.99 (0.91 - 1.08) | 0.82 | 1.09 (0.96 - 1.23) | 0.20 | 1.07 (0.94 - 1.22) | 0.33 |
| Sleep health score | 1.03 (0.97 - 1.09) | 0.33 | 1.01 (0.96 - 1.08) | 0.65 | 1.01 (0.92 - 1.11) | 0.84 | 1.00 (0.91 - 1.10) | 0.96 |

**Bold** cells denote P<0.05.

^1^Sociodemographic factors include education, occupational status, and marital status.

^2^Behavioral factors consist of alcohol risk consumption, physical activity, and smoking status.

^3^Health-related factors include current depressive symptomatology, use of hypnotics and sedatives, body mass index, prevalent diabetes, and prevalent cardiovascular diseases.

**eTable 11.** Association of phenotypic sleep traits with incident dementia within complete twin pairs in the total sample with available self-reported sleep traits (co-twin control analysis)

| **Phenotypic traits** | **Adjusted for sex** | | **Additionally Adjusted for Sociodemographic Factors^1^** | | **Additionally Adjusted for Behavioral Factors^2^** | | **Additionally Adjusted for Health-Related Factors^3^** | |
| --- | --- | --- | --- | --- | --- | --- | --- | --- |
|  | **HR (95% CI)** | **P** | **HR (95% CI)** | **P** | **HR (95% CI)** | **P** | **HR (95% CI)** | **P** |
| Sleep duration | 1.06 (0.95 - 1.17) | 0.28 | 1.02 (0.91 - 1.15) | 0.73 | 1.02 (0.86 - 1.20) | 0.82 | 1.08 (0.89 - 1.31) | 0.43 |
| Chronotype | **1.11 (1.03 - 1.19)** | **0.005** | **1.12 (1.04 - 1.22)** | **0.004** | **1.13 (1.01 - 1.27)** | **0.03** | 1.07 (0.94 - 1.21) | 0.29 |
| Insomnia | 0.74 (0.54 - 1.02) | 0.06 | 0.69 (0.48 - 1.00) | 0.05 | 0.59 (0.33 - 1.05) | 0.07 | 0.66 (0.35 - 1.24) | 0.19 |
| Snoring | 0.98 (0.72 - 1.35) | 0.92 | 1.02 (0.71 - 1.45) | 0.93 | 0.98 (0.56 - 1.71) | 0.95 | 1.12 (0.61 - 2.07) | 0.71 |
| Daytime sleepiness | 0.97 (0.79 - 1.19) | 0.77 | 0.89 (0.71 - 1.11) | 0.30 | 0.73 (0.51 - 1.06) | 0.10 | 0.80 (0.54 - 1.19) | 0.27 |
| Daytime napping | 1.14 (0.93 - 1.39) | 0.21 | 1.13 (0.90 - 1.41) | 0.30 | 1.14 (0.84 - 1.54) | 0.40 | 1.00 (0.71 - 1.43) | 0.98 |
| Sleep health score | 1.06 (0.91 - 1.24) | 0.45 | 1.07 (0.90 - 1.28) | 0.45 | 1.26 (0.97 - 1.65) | 0.09 | 1.14 (0.86 - 1.52) | 0.36 |

**Bold** cells denote P<0.05.

^1^Sociodemographic factors include education, occupational status, and marital status.

^2^Behavioral factors consist of alcohol risk consumption, physical activity, and smoking status.

^3^Health-related factors include current depressive symptomatology, use of hypnotics and sedatives, body mass index, prevalent diabetes, and prevalent cardiovascular diseases.

**eTable 12.** Association of PGS for midlife sleep traits and dementia risk in the genotyped sample

| **Polygenic score (z-score)** | **Adjusted for sex and study** | | **Additionally Adjusted for Sociodemographic Factors^1^** | | **Additionally Adjusted for Behavioral Factors^2^** | | **Additionally Adjusted for Health-Related Factors^3^** | |
| --- | --- | --- | --- | --- | --- | --- | --- | --- |
|  | **HR (95% CI)** | **P** | **HR (95% CI)** | **P** | **HR (95% CI)** | **P** | **HR (95% CI)** | **P** |
| Sleep duration | 1.03 (0.95 - 1.12) | 0.41 | 1.05 (0.97 - 1.15) | 0.24 | 1.08 (0.98 - 1.19) | 0.13 | 1.08 (0.97 - 1.19) | 0.15 |
| Chronotype | 1.02 (0.94 - 1.11) | 0.59 | 1.01 (0.93 - 1.10) | 0.77 | 0.99 (0.90 - 1.09) | 0.83 | 1.01 (0.92 - 1.12) | 0.78 |
| Insomnia | 0.97 (0.90 - 1.05) | 0.41 | 0.97 (0.90 - 1.05) | 0.42 | 0.97 (0.89 - 1.07) | 0.55 | 0.99 (0.90 - 1.08) | 0.77 |
| Snoring | 1.03 (0.96 - 1.11) | 0.39 | 1.03 (0.96 - 1.12) | 0.41 | 1.02 (0.93 - 1.12) | 0.67 | 1.02 (0.93 - 1.11) | 0.73 |
| Daytime sleepiness | 1.04 (0.96 - 1.12) | 0.31 | 1.04 (0.96 - 1.13) | 0.32 | 1.06 (0.96 - 1.16) | 0.24 | 1.06 (0.96 - 1.16) | 0.25 |
| Daytime napping | **1.11 (1.03 - 1.20)** | **0.005** | **1.12 (1.04 - 1.21)** | **0.003** | **1.11 (1.01 - 1.21)** | **0.03** | **1.11 (1.01 - 1.21)** | **0.03** |
| Sleep health score | 0.99 (0.91 - 1.07) | 0.73 | 0.99 (0.91 - 1.07) | 0.73 | 0.99 (0.91 - 1.08) | 0.81 | 1.00 (0.91 - 1.09) | 0.92 |

**Bold** cells denote P<0.05.

^1^Sociodemographic factors include education, occupational status, and marital status.

^2^Behavioral factors consist of alcohol risk consumption, physical activity, and smoking status.

^3^Health-related factors include current depressive symptomatology, use of hypnotics and sedatives, body mass index, prevalent diabetes, and prevalent cardiovascular diseases.

**eTable 13.** Marginal and joint effect associations of phenotypic sleep traits and their corresponding PGS with dementia risk in subsample with both phenotypic and genotyped data available.

|  | **Adjusted for sex and study^1^** | | **Additionally Adjusted for Sociodemographic Factors^2^** | | **Additionally Adjusted for Behavioral Factors^3^** | | **Additionally Adjusted for Health-Related Factors^4^** | |
| --- | --- | --- | --- | --- | --- | --- | --- | --- |
|  | **HR (95% CI)** | **P** | **HR (95% CI)** | **P** | **HR (95% CI)** | **P** | **HR (95% CI)** | **P** |
| **Marginal effect model** |  |  |  |  |  |  |  |  |
| **Phenotypic trait** |  |  |  |  |  |  |  |  |
| Sleep duration | 0.98 (0.88 - 1.08) | 0.62 | 0.96 (0.87 - 1.07) | 0.45 | 0.91 (0.80 - 1.04) | 0.17 | 0.93 (0.81 - 1.06) | 0.28 |
| Chronotype | 1.02 (0.95 - 1.08) | 0.59 | 1.01 (0.94 - 1.07) | 0.85 | 0.97 (0.89 - 1.05) | 0.48 | 0.98 (0.91 - 1.07) | 0.72 |
| Insomnia | 1.11 (0.80 - 1.54) | 0.53 | 1.17 (0.83 - 1.65) | 0.36 | 1.28 (0.82 - 2.00) | 0.28 | 1.17 (0.72 - 1.89) | 0.53 |
| Snoring | 1.02 (0.77 - 1.34) | 0.91 | 1.00 (0.75 - 1.33) | 0.99 | 0.94 (0.65 - 1.37) | 0.77 | 0.95 (0.64 - 1.41) | 0.81 |
| Daytime sleepiness | 1.01 (0.82 - 1.26) | 0.91 | 1.02 (0.82 - 1.27) | 0.87 | 1.22 (0.92 - 1.61) | 0.16 | 1.22 (0.92 - 1.61) | 0.16 |
| Daytime napping | 1.08 (0.90 - 1.30) | 0.40 | 1.09 (0.90 - 1.32) | 0.37 | 1.14 (0.90 - 1.44) | 0.29 | 1.11 (0.87 - 1.42) | 0.41 |
| Sleep health score | 0.99 (0.86 - 1.15) | 0.92 | 0.98 (0.85 - 1.14) | 0.83 | 1.02 (0.83 - 1.24) | 0.86 | 1.03 (0.85 - 1.26) | 0.75 |
| **Polygenic score (z-score)** |  |  |  |  |  |  |  |  |
| Sleep duration | 1.01 (0.92 - 1.11) | 0.81 | 1.02 (0.92 - 1.13) | 0.67 | 1.07 (0.95 - 1.21) | 0.26 | 1.09 (0.97 - 1.24) | 0.16 |
| Chronotype | 1.05 (0.95 - 1.16) | 0.31 | 1.03 (0.94 - 1.14) | 0.50 | 0.99 (0.88 - 1.11) | 0.84 | 1.01 (0.90 - 1.15) | 0.82 |
| Insomnia | 0.96 (0.85 - 1.09) | 0.51 | 0.96 (0.84 - 1.09) | 0.55 | 0.95 (0.81 - 1.12) | 0.56 | 0.94 (0.79 - 1.12) | 0.50 |
| Snoring | 1.09 (0.94 - 1.26) | 0.26 | 1.12 (0.96 - 1.31) | 0.14 | 1.09 (0.89 - 1.34) | 0.41 | 1.08 (0.88 - 1.33) | 0.46 |
| Daytime sleepiness | 1.07 (0.94 - 1.21) | 0.32 | 1.08 (0.95 - 1.23) | 0.25 | 1.14 (0.96 - 1.35) | 0.13 | 1.12 (0.94 - 1.33) | 0.20 |
| Daytime napping | **1.11 (1.02 - 1.21)** | **0.02** | **1.13 (1.03 - 1.24)** | **0.01** | **1.13 (1.01 - 1.27)** | **0.04** | **1.13 (1.01 - 1.27)** | **0.04** |
| Sleep health score | 0.96 (0.83 - 1.11) | 0.57 | 0.93 (0.80 - 1.08) | 0.34 | 0.94 (0.79 - 1.12) | 0.48 | 0.95 (0.80 - 1.13) | 0.54 |
| **Joint effect model** |  |  |  |  |  |  |  |  |
| **Phenotypic trait** |  |  |  |  |  |  |  |  |
| Sleep duration | 0.98 (0.88 - 1.08) | 0.62 | 0.96 (0.87 - 1.06) | 0.44 | 0.91 (0.79 - 1.04) | 0.15 | 0.92 (0.80 - 1.06) | 0.25 |
| Chronotype | 1.01 (0.95 - 1.08) | 0.76 | 1.00 (0.94 - 1.07) | 0.99 | 0.97 (0.89 - 1.06) | 0.50 | 0.98 (0.90 - 1.07) | 0.67 |
| Insomnia | 1.12 (0.80 - 1.55) | 0.52 | 1.17 (0.84 - 1.65) | 0.36 | 1.29 (0.82 - 2.01) | 0.27 | 1.18 (0.72 - 1.91) | 0.51 |
| Snoring | 0.99 (0.75 - 1.31) | 0.94 | 0.97 (0.73 - 1.29) | 0.83 | 0.93 (0.64 - 1.35) | 0.69 | 0.94 (0.63 - 1.39) | 0.75 |
| Daytime sleepiness | 1.01 (0.82 - 1.26) | 0.90 | 1.02 (0.82 - 1.27) | 0.86 | 1.20 (0.92 - 1.58) | 0.18 | 1.20 (0.91 - 1.58) | 0.19 |
| Daytime napping | 1.05 (0.87 - 1.26) | 0.61 | 1.06 (0.87 - 1.28) | 0.58 | 1.10 (0.87 - 1.40) | 0.41 | 1.07 (0.84 - 1.38) | 0.57 |
| Sleep health score | 1.00 (0.86 - 1.15) | 0.95 | 0.99 (0.86 - 1.14) | 0.87 | 1.02 (0.84 - 1.25) | 0.81 | 1.04 (0.85 - 1.26) | 0.71 |
| **Polygenic score (z-score)** |  |  |  |  |  |  |  |  |
| Sleep duration | 1.01 (0.92 - 1.12) | 0.78 | 1.02 (0.93 - 1.13) | 0.64 | 1.08 (0.95 - 1.21) | 0.23 | 1.10 (0.97 - 1.25) | 0.14 |
| Chronotype | 1.05 (0.95 - 1.16) | 0.36 | 1.03 (0.93 - 1.15) | 0.52 | 1.00 (0.88 - 1.13) | 0.98 | s1.02 (0.90 - 1.16) | 0.74 |
| Insomnia | 0.96 (0.84 - 1.09) | 0.50 | 0.96 (0.84 - 1.09) | 0.54 | 0.95 (0.80 - 1.12) | 0.53 | 0.94 (0.79 - 1.12) | 0.48 |
| Snoring | 1.09 (0.94 - 1.26) | 0.26 | 1.12 (0.96 - 1.31) | 0.13 | 1.09 (0.89 - 1.35) | 0.39 | 1.08 (0.88 - 1.33) | 0.45 |
| Daytime sleepiness | 1.07 (0.94 - 1.21) | 0.32 | 1.08 (0.95 - 1.23) | 0.25 | 1.13 (0.96 - 1.35) | 0.15 | 1.11 (0.93 - 1.32) | 0.24 |
| Daytime napping | **1.11 (1.01 - 1.21)** | **0.02** | **1.13 (1.03 - 1.23)** | **0.01** | **1.13 (1.00 - 1.27)** | **0.047** | **1.13 (1.00 - 1.27)** | **0.046** |
| Sleep health score | 0.96 (0.83 - 1.11) | 0.58 | 0.93 (0.80 - 1.08) | 0.35 | 0.94 (0.79 - 1.12) | 0.48 | 0.95 (0.79 - 1.13) | 0.53 |

**Bold** cells denote P<0.05.

^1^For independent association of phenotypic sleep traits, model was adjusted only for sex.

^2^Sociodemographic factors include education, occupational status, and marital status.

^3^Behavioral factors consist of alcohol risk consumption, physical activity, and smoking status.

^4^Health-related factors include current depressive symptomatology, use of hypnotics and sedatives, body mass index, prevalent diabetes, and prevalent cardiovascular diseases.

**eTable 14**. Main and interactive effect terms of phenotypic sleep traits and their corresponding PGS with dementia risk excluding dementia cases occurring within the first 10 years of follow-up.

|  | **Adjusted for sex and study^1^** | | **Additionally Adjusted for Sociodemographic Factors^2^** | | **Additionally Adjusted for Behavioral Factors^3^** | | **Additionally Adjusted for Health-Related Factors^4^** | |
| --- | --- | --- | --- | --- | --- | --- | --- | --- |
|  | **HR (95% CI)** | **P** | **HR (95% CI)** | **P** | **HR (95% CI)** | **P** | **HR (95% CI)** | **P** |
| **Sleep duration** |  |  |  |  |  |  |  |  |
| **Main effect** |  |  |  |  |  |  |  |  |
| Phenotypic | 0.94 (0.84 - 1.05) | 0.26 | 0.93 (0.83 - 1.04) | 0.20 | 0.85 (0.74 - 0.99) | 0.03 | 0.88 (0.75 - 1.03) | 0.11 |
| PGS | 1.55 (0.59 - 4.07) | 0.38 | 1.54 (0.56 - 4.18) | 0.40 | 1.54 (0.48 - 5.08) | 0.46 | 2.19 (0.57 - 8.38) | 0.25 |
| **Interaction term** |  |  |  |  |  |  |  |  |
| Phenotypic*PGS | 0.95 (0.85 - 1.07) | 0.40 | 0.96 (0.85 - 1.07) | 0.45 | 0.96 (0.83 - 1.10) | 0.56 | 0.92 (0.79 - 1.08) | 0.33 |
| **Chronotype** |  |  |  |  |  |  |  |  |
| **Main effect** |  |  |  |  |  |  |  |  |
| Phenotypic | 1.02 (0.94 - 1.09) | 0.69 | 1.00 (0.93 - 1.08) | 0.95 | 0.97 (0.88 - 1.08) | 0.62 | 0.99 (0.89 - 1.09) | 0.77 |
| PGS | 1.09 (0.96 - 1.23) | 0.17 | 1.07 (0.95 - 1.21) | 0.27 | 1.07 (0.89 - 1.19) | 0.70 | 1.05 (0.91 - 1.22) | 0.51 |
| **Interaction term** |  |  |  |  |  |  |  |  |
| Phenotypic*PGS | 1.01 (0.94 - 1.09) | 0.78 | 1.00 (0.93 - 1.09) | 0.91 | 0.99 (0.90 - 1.10) | 0.89 | 1.01 (0.91 - 1.11) | 0.90 |
| **Insomnia** |  |  |  |  |  |  |  |  |
| **Main effect** |  |  |  |  |  |  |  |  |
| Phenotypic | 1.15 (0.81 - 1.63) | 0.44 | 1.20 (0.84 - 1.73) | 0.32 | 1.30 (0.80 - 2.12) | 0.29 | 1.22 (0.72 - 2.07) | 0.47 |
| PGS | 0.94 (0.80 - 1.09) | 0.40 | 0.92 (0.78 - 1.08) | 0.32 | 0.92 (0.74 - 1.13) | 0.42 | 0.90 (0.72 - 1.12) | 0.35 |
| **Interaction term** |  |  |  |  |  |  |  |  |
| Phenotypic*PGS | 1.08 (0.80 - 1.45) | 0.63 | 1.15 (0.85 - 1.55) | 0.37 | 1.08 (0.77 - 1.52) | 0.64 | 1.08 (0.75 - 1.55) | 0.69 |
| **Snoring** |  |  |  |  |  |  |  |  |
| **Main effect** |  |  |  |  |  |  |  |  |
| Phenotypic | 1.03 (0.76 - 1.41) | 0.83 | 1.01 (0.74 - 1.39) | 0.93 | 0.96 (0.63 - 1.45) | 0.83 | 0.98 (0.63 - 1.53) | 0.94 |
| PGS | 1.05 (0.85 - 1.30) | 0.66 | 1.06 (0.86 - 1.32) | 0.58 | 1.06 (0.68 - 1.30) | 0.70 | 0.93 (0.68 - 1.28) | 0.66 |
| **Interaction term** |  |  |  |  |  |  |  |  |
| Phenotypic*PGS | 1.04 (0.76 - 1.42) | 0.80 | 1.10 (0.80 - 1.50) | 0.56 | 1.24 (0.80 - 1.90) | 0.33 | 1.23 (0.79 - 1.91) | 0.37 |
| **Daytime sleepiness** |  |  |  |  |  |  |  |  |
| **Main effect** |  |  |  |  |  |  |  |  |
| Phenotypic | 1.02 (0.81 - 1.28) | 0.87 | 1.04 (0.82 - 1.31) | 0.76 | 1.23 (0.89 - 1.69) | 0.21 | 1.24 (0.90 - 1.71) | 0.19 |
| PGS | 0.79 (0.57 - 1.09) | 0.15 | 0.86 (0.61 - 1.20) | 0.37 | 0.86 (0.69 - 1.54) | 0.89 | 1.08 (0.71 - 1.66) | 0.71 |
| **Interaction term** |  |  |  |  |  |  |  |  |
| Phenotypic*PGS | 1.23 (1.00 - 1.52) | 0.05 | 1.18 (0.95 - 1.46) | 0.14 | 1.08 (0.84 - 1.38) | 0.55 | 1.02 (0.78 - 1.34) | 0.87 |
| **Daytime napping** |  |  |  |  |  |  |  |  |
| **Main effect** |  |  |  |  |  |  |  |  |
| Phenotypic | 1.00 (0.81 - 1.23) | 0.99 | 1.00 (0.81 - 1.24) | 0.99 | 1.02 (0.77 - 1.33) | 0.91 | 1.01 (0.76 - 1.34) | 0.95 |
| PGS | 1.10 (0.96 - 1.25) | 0.17 | 1.11 (0.96 - 1.27) | 0.16 | 1.11 (0.93 - 1.32) | 0.27 | 1.12 (0.94 - 1.34) | 0.22 |
| **Interaction term** |  |  |  |  |  |  |  |  |
| Phenotypic*PGS | 1.06 (0.87 - 1.29) | 0.54 | 1.09 (0.89 - 1.33) | 0.41 | 1.16 (0.90 - 1.49) | 0.26 | 1.12 (0.86 - 1.45) | 0.41 |
| **Sleep Health Score** |  |  |  |  |  |  |  |  |
| **Main effect** |  |  |  |  |  |  |  |  |
| Phenotypic | 0.99 (0.84 - 1.15) | 0.87 | 0.97 (0.83 - 1.14) | 0.75 | 1.01 (0.81 - 1.26) | 0.93 | 1.02 (0.82 - 1.27) | 0.88 |
| PGS | 0.82 (0.50 - 1.35) | 0.44 | 0.86 (0.52 - 1.44) | 0.57 | 0.86 (0.54 - 1.84) | 1.00 | 0.97 (0.52 - 1.80) | 0.92 |
| **Interaction term** |  |  |  |  |  |  |  |  |
| Phenotypic*PGS | 1.06 (0.90 - 1.25) | 0.47 | 1.03 (0.88 - 1.22) | 0.69 | 0.98 (0.80 - 1.22) | 0.88 | 1.00 (0.80 - 1.25) | 0.99 |

**Bold** cells denote P<0.05.

^1^For independent association of phenotypic sleep traits, model was adjusted only for sex.

^2^Sociodemographic factors include education, occupational status, and marital status.

^3^Behavioral factors consist of alcohol risk consumption, physical activity, and smoking status.

^4^Health-related factors include current depressive symptomatology, use of hypnotics and sedatives, body mass index, prevalent diabetes, and prevalent cardiovascular diseases.

**eTable 15.** Main and interactive effect terms of phenotypic sleep traits and their corresponding PGS with dementia risk

|  | **Adjusted for sex and study^1^** | | **Additionally Adjusted for Sociodemographic Factors^2^** | | **Additionally Adjusted for Behavioral Factors^3^** | | **Additionally Adjusted for Health-Related Factors^4^** | |
| --- | --- | --- | --- | --- | --- | --- | --- | --- |
|  | **HR (95% CI)** | **P** | **HR (95% CI)** | **P** | **HR (95% CI)** | **P** | **HR (95% CI)** | **P** |
| **Sleep duration** |  |  |  |  |  |  |  |  |
| **Main effect** |  |  |  |  |  |  |  |  |
| Phenotypic | 0.98 (0.88 - 1.08) | 0.63 | 0.96 (0.87 - 1.07) | 0.45 | 0.91 (0.79 - 1.04) | 0.15 | 0.92 (0.80 - 1.06) | 0.25 |
| PGS | 1.30 (0.53 - 3.19) | 0.57 | 1.37 (0.54 - 3.51) | 0.51 | 1.37 (0.35 - 3.43) | 0.88 | 1.32 (0.36 - 4.87) | 0.68 |
| **Interaction term** |  |  |  |  |  |  |  |  |
| Phenotypic*PGS | 0.97 (0.87 - 1.08) | 0.58 | 0.97 (0.87 - 1.08) | 0.53 | 1.00 (0.87 - 1.14) | 0.98 | 0.98 (0.84 - 1.14) | 0.78 |
| **Chronotype** |  |  |  |  |  |  |  |  |
| **Main effect** |  |  |  |  |  |  |  |  |
| Phenotypic | 1.01 (0.95 - 1.08) | 0.76 | 1.00 (0.93 - 1.07) | 1.00 | 0.97 (0.89 - 1.06) | 0.50 | 0.98 (0.90 - 1.07) | 0.69 |
| PGS | 1.05 (0.94 - 1.17) | 0.40 | 1.04 (0.93 - 1.16) | 0.53 | 1.04 (0.88 - 1.14) | 1.00 | 1.02 (0.89 - 1.17) | 0.78 |
| **Interaction term** |  |  |  |  |  |  |  |  |
| Phenotypic*PGS | 1.00 (0.93 - 1.07) | 0.99 | 1.00 (0.93 - 1.07) | 0.89 | 1.00 (0.91 - 1.09) | 0.92 | 1.01 (0.92 - 1.10) | 0.91 |
| **Insomnia** |  |  |  |  |  |  |  |  |
| **Main effect** |  |  |  |  |  |  |  |  |
| Phenotypic | 1.12 (0.80 - 1.55) | 0.52 | 1.18 (0.84 - 1.65) | 0.35 | 1.28 (0.82 - 2.01) | 0.27 | 1.18 (0.72 - 1.91) | 0.51 |
| PGS | 0.95 (0.83 - 1.10) | 0.50 | 0.94 (0.81 - 1.09) | 0.44 | 0.94 (0.78 - 1.16) | 0.62 | 0.94 (0.76 - 1.15) | 0.55 |
| **Interaction term** |  |  |  |  |  |  |  |  |
| Phenotypic*PGS | 1.03 (0.78 - 1.35) | 0.83 | 1.09 (0.82 - 1.44) | 0.56 | 0.98 (0.72 - 1.35) | 0.91 | 1.00 (0.72 - 1.39) | 0.99 |
| **Snoring** |  |  |  |  |  |  |  |  |
| **Main effect** |  |  |  |  |  |  |  |  |
| Phenotypic | 0.99 (0.75 - 1.31) | 0.94 | 0.97 (0.73 - 1.29) | 0.82 | 0.92 (0.63 - 1.35) | 0.68 | 0.94 (0.63 - 1.39) | 0.74 |
| PGS | 1.07 (0.89 - 1.30) | 0.46 | 1.09 (0.89 - 1.32) | 0.42 | 1.09 (0.76 - 1.36) | 0.93 | 1.00 (0.75 - 1.34) | 0.98 |
| **Interaction term** |  |  |  |  |  |  |  |  |
| Phenotypic*PGS | 1.03 (0.76 - 1.38) | 0.86 | 1.07 (0.79 - 1.45) | 0.65 | 1.15 (0.76 - 1.73) | 0.52 | 1.14 (0.75 - 1.74) | 0.53 |
| **Daytime sleepiness** |  |  |  |  |  |  |  |  |
| **Main effect** |  |  |  |  |  |  |  |  |
| Phenotypic | 0.97 (0.78 - 1.20) | 0.75 | 0.98 (0.79 - 1.22) | 0.85 | 1.15 (0.86 - 1.54) | 0.36 | 1.16 (0.86 - 1.56) | 0.33 |
| PGS | 0.78 (0.59 - 1.04) | 0.09 | 0.84 (0.62 - 1.13) | 0.25 | 0.84 (0.66 - 1.36) | 0.78 | 0.99 (0.68 - 1.45) | 0.96 |
| **Interaction term** |  |  |  |  |  |  |  |  |
| Phenotypic*PGS | **1.26 (1.05 - 1.51)** | **0.01** | 1.21 (1.00 - 1.46) | 0.054 | 1.14 (0.92 - 1.42) | 0.23 | 1.09 (0.86 - 1.38) | 0.47 |
| **Daytime napping** |  |  |  |  |  |  |  |  |
| **Main effect** |  |  |  |  |  |  |  |  |
| Phenotypic | 1.04 (0.86 - 1.25) | 0.70 | 1.04 (0.86 - 1.26) | 0.68 | 1.08 (0.85 - 1.38) | 0.52 | 1.06 (0.82 - 1.37) | 0.64 |
| PGS | 1.06 (0.95 - 1.20) | 0.30 | 1.08 (0.95 - 1.22) | 0.24 | 1.08 (0.91 - 1.25) | 0.46 | 1.09 (0.93 - 1.28) | 0.30 |
| **Interaction term** |  |  |  |  |  |  |  |  |
| Phenotypic*PGS | 1.08 (0.91 - 1.29) | 0.36 | 1.10 (0.92 - 1.31) | 0.31 | 1.14 (0.90 - 1.43) | 0.27 | 1.08 (0.86 - 1.36) | 0.52 |
| **Sleep Health Score** |  |  |  |  |  |  |  |  |
| **Main effect** |  |  |  |  |  |  |  |  |
| Phenotypic | 0.99 (0.86 - 1.15) | 0.94 | 0.99 (0.85 - 1.14) | 0.86 | 1.03 (0.84 - 1.25) | 0.81 | 1.04 (0.85 - 1.26) | 0.71 |
| PGS | 0.77 (0.49 - 1.22) | 0.27 | 0.80 (0.50 - 1.28) | 0.36 | 0.80 (0.50 - 1.58) | 0.69 | 0.87 (0.49 - 1.54) | 0.63 |
| **Interaction term** |  |  |  |  |  |  |  |  |
| Phenotypic*PGS | 1.07 (0.92 - 1.25) | 0.35 | 1.05 (0.90 - 1.23) | 0.53 | 1.02 (0.83 - 1.24) | 0.86 | 1.03 (0.84 - 1.26) | 0.78 |

**Bold** cells denote P<0.05.

^1^For independent association of phenotypic sleep traits, model was adjusted only for sex.

^2^Sociodemographic factors include education, occupational status, and marital status.

^3^Behavioral factors consist of alcohol risk consumption, physical activity, and smoking status.

^4^Health-related factors include current depressive symptomatology, use of hypnotics and sedatives, body mass index, prevalent diabetes, and prevalent cardiovascular diseases.

**eTable 16.** Post hoc sensitivity analyses of the PGS for midlife daytime napping stratified by APOE ε4 allele carrier status

| **PGS for midlife daytime napping** | **Non *APOE*** **ε4 allele carrier** | | ***APOE*** **ε4 allele carriers** | |
| --- | --- | --- | --- | --- |
|  | **HR (95% CI)** | **P** | **HR (95% CI)** | **P** |
|  |  |  |  |  |
| **Total Genotyped data** |  |  |  |  |
| Unrestricted sample | **1.20 (1.04 - 1.37)** | **0.01** | 1.04 (0.92 - 1.18) | 0.522 |
| Restricted sample | **1.20 (1.03 - 1.39)** | **0.02** | 1.05 (0.92 - 1.21) | 0.439 |
|  |  |  |  |  |
| **Combined phenotypic and genotyped data** |  |  |  |  |
| ***Unrestricted sample*** |  |  |  |  |
| Marginal effect | 1.17 (0.99 - 1.39) | 0.069 | 1.10 (0.94 - 1.29) | 0.223 |
| Joint effect | 1.18 (0.99 - 1.40) | 0.061 | 1.09 (0.93 - 1.28) | 0.273 |
| ***Restricted sample*** |  |  |  |  |
| Marginal effect | 1.18 (0.98 – 1.42) | 0.085 | **1.20 (1.01 - 1.44)** | **0.038** |
| Joint effect | 1.19 (0.99 – 1.43) | 0.069 | **1.19 (1.00 – 1.43)** | **0.050** |

**Bold** cells denote P<0.05. The restricted sample refers to participants who received a dementia diagnosis within the first 10 years of follow‑up, whereas the unrestricted sample includes all eligible participants.

All models are adjusted for sex, study, sociodemographic (education, occupational status, and marital status), behavioural (alcohol risk consumption, physical activity, and smoking status), and health-related factors (current depressive symptomatology, use of hypnotics and sedatives, body mass index, prevalent diabetes, and prevalent cardiovascular diseases).

**eTable 17.** Association of phenotypic sleep traits with incident dementia in the total sample with available self-reported sleep traits excluding dementia cases occurring within the first 10 years of follow-up using competing-risk analyses

| **Phenotypic traits** | **Adjusted for sex** | | **Additionally Adjusted for Sociodemographic Factors^1^** | | **Additionally Adjusted for Behavioral Factors^2^** | | **Additionally Adjusted for Health-Related Factors^3^** | |
| --- | --- | --- | --- | --- | --- | --- | --- | --- |
|  | **HR (95% CI)** | **P** | **HR (95% CI)** | **P** | **HR (95% CI)** | **P** | **HR (95% CI)** | **P** |
| Sleep duration | **0.94 (0.90 - 0.99)** | **0.01** | **0.93 (0.89 - 0.98)** | **0.01** | 0.95 (0.89 - 1.03) | 0.21 | 0.96 (0.89 - 1.03) | 0.27 |
| Chronotype | 1.02 (0.99 - 1.06) | 0.21 | 1.02 (0.98 - 1.06) | 0.31 | 1.00 (0.95 - 1.06) | 0.89 | 1.00 (0.95 - 1.06) | 0.90 |
| Insomnia | 1.06 (0.90 - 1.25) | 0.49 | 1.12 (0.93 - 1.34) | 0.23 | 1.14 (0.87 - 1.48) | 0.35 | 1.13 (0.85 - 1.50) | 0.39 |
| Snoring | 1.11 (0.96 - 1.29) | 0.16 | 1.08 (0.92 - 1.26) | 0.37 | 1.10 (0.87 - 1.37) | 0.43 | 1.19 (0.94 - 1.51) | 0.15 |
| Daytime sleepiness | 0.92 (0.83 - 1.03) | 0.17 | 0.96 (0.85 - 1.07) | 0.46 | 1.04 (0.87 - 1.24) | 0.67 | 1.06 (0.89 - 1.27) | 0.49 |
| Daytime napping | 0.91 (0.82 - 1.01) | 0.07 | 0.91 (0.81 - 1.01) | 0.08 | 1.01 (0.86 - 1.17) | 0.93 | 1.03 (0.88 - 1.21) | 0.67 |
| Sleep health score | 1.00 (0.93 - 1.07) | 0.95 | 0.98 (0.91 - 1.06) | 0.63 | 0.98 (0.88 - 1.09) | 0.70 | 0.95 (0.86 - 1.06) | 0.37 |

**Bold** cells denote P<0.05.

^1^Sociodemographic factors include education, occupational status, and marital status.

^2^Behavioral factors consist of alcohol risk consumption, physical activity, and smoking status.

^3^Health-related factors include current depressive symptomatology, use of hypnotics and sedatives, body mass index, prevalent diabetes, and prevalent cardiovascular diseases.

**eTable 18.** Association of PGS for midlife sleep traits and dementia risk in the genotyped sample excluding dementia cases occurring within the first 10 years of follow-up using competing-risk analyses

| **Polygenic score (z-score)** | **Adjusted for sex and study^1^** | | **Additionally Adjusted for Sociodemographic Factors^2^** | | **Additionally Adjusted for Behavioral Factors^3^** | | **Additionally Adjusted for Health-Related Factors^4^** | |
| --- | --- | --- | --- | --- | --- | --- | --- | --- |
|  | **HR (95% CI)** | **P** | **HR (95% CI)** | **P** | **HR (95% CI)** | **P** | **HR (95% CI)** | **P** |
| Sleep duration | 1.03 (0.94 - 1.13) | 0.49 | 1.06 (0.96 - 1.16) | 0.24 | 1.09 (0.98 - 1.21) | 0.11 | 1.08 (0.97 - 1.21) | 0.16 |
| Chronotype | 1.04 (0.95 - 1.13) | 0.45 | 1.01 (0.93 - 1.11) | 0.75 | 1.00 (0.90 - 1.11) | 0.97 | 1.02 (0.92 - 1.14) | 0.70 |
| Insomnia | 0.97 (0.89 - 1.05) | 0.43 | 0.96 (0.88 - 1.05) | 0.39 | 0.94 (0.85 - 1.04) | 0.26 | 0.96 (0.87 - 1.07) | 0.48 |
| Snoring | 0.99 (0.91 - 1.08) | 0.85 | 0.99 (0.91 - 1.08) | 0.85 | 0.99 (0.90 - 1.09) | 0.84 | 0.99 (0.89 - 1.09) | 0.80 |
| Daytime sleepiness | 1.05 (0.96 - 1.15) | 0.26 | 1.05 (0.96 - 1.15) | 0.27 | 1.07 (0.97 - 1.19) | 0.19 | 1.08 (0.97 - 1.20) | 0.15 |
| Daytime napping | **1.11 (1.02 - 1.21)** | **0.01** | **1.12 (1.03 - 1.22)** | **0.01** | **1.12 (1.01 - 1.24)** | **0.03** | **1.11 (1.01 - 1.23)** | **0.04** |
| Sleep health score | 1.01 (0.93 - 1.10) | 0.84 | 1.01 (0.92 - 1.10) | 0.87 | 1.02 (0.92 - 1.12) | 0.76 | 1.01 (0.92 - 1.12) | 0.83 |

**Bold** cells denote P<0.05.^1^Sociodemographic factors include education, occupational status, and marital status. ^2^Behavioral factors consist of alcohol risk consumption, physical activity, and smoking status. ^3^Health-related factors include current depressive symptomatology, use of hypnotics and sedatives, body mass index, prevalent diabetes, and prevalent cardiovascular diseases.

**eTable 19.** Marginal and joint effect associations of phenotypic sleep traits and their corresponding midlife PGS with dementia risk excluding dementia cases occurring within the first 10 years of follow-up using competing-risk analyses.

|  | **Adjusted for sex and study^1^** | | **Additionally Adjusted for Sociodemographic Factors^2^** | | **Additionally Adjusted for Behavioral Factors^3^** | | **Additionally Adjusted for Health-Related Factors^4^** | |
| --- | --- | --- | --- | --- | --- | --- | --- | --- |
|  | **HR (95% CI)** | **P** | **HR (95% CI)** | **P** | **HR (95% CI)** | **P** | **HR (95% CI)** | **P** |
| **Marginal effect model** |  |  |  |  |  |  |  |  |
| **Phenotypic trait** |  |  |  |  |  |  |  |  |
| Sleep duration | 0.98 (0.87 - 1.09) | 0.66 | 0.96 (0.86 - 1.08) | 0.52 | 0.89 (0.77 - 1.03) | 0.12 | 0.90 (0.78 - 1.05) | 0.19 |
| Chronotype | 1.03 (0.95 - 1.11) | 0.48 | 1.02 (0.94 - 1.09) | 0.70 | 0.99 (0.90 - 1.09) | 0.84 | 0.99 (0.90 - 1.09) | 0.90 |
| Insomnia | 1.36 (0.97 - 1.91) | 0.07 | **1.44 (1.02 - 2.03)** | **0.04** | 1.39 (0.87 - 2.23) | 0.17 | 1.36 (0.82 - 2.23) | 0.23 |
| Snoring | 1.03 (0.75 - 1.42) | 0.85 | 1.00 (0.72 - 1.39) | 0.99 | 1.01 (0.67 - 1.52) | 0.97 | 1.09 (0.70 - 1.68) | 0.71 |
| Daytime sleepiness | 1.09 (0.87 - 1.37) | 0.47 | 1.11 (0.88 - 1.40) | 0.38 | 1.24 (0.90 - 1.72) | 0.19 | 1.25 (0.90 - 1.73) | 0.19 |
| Daytime napping | 1.13 (0.92 - 1.38) | 0.25 | 1.12 (0.91 - 1.39) | 0.28 | 1.09 (0.84 - 1.43) | 0.50 | 1.12 (0.85 - 1.47) | 0.43 |
| Sleep health score | 0.96 (0.82 - 1.12) | 0.63 | 0.95 (0.82 - 1.11) | 0.54 | 0.99 (0.80 - 1.22) | 0.94 | 0.97 (0.78 - 1.20) | 0.79 |
| **Polygenic score (z-score)** |  |  |  |  |  |  |  |  |
| Sleep duration | 1.00 (0.90 - 1.12) | 0.93 | 1.03 (0.92 - 1.15) | 0.66 | 1.10 (0.96 - 1.25) | 0.18 | 1.11 (0.97 - 1.28) | 0.13 |
| Chronotype | 1.08 (0.97 - 1.20) | 0.14 | 1.05 (0.94 - 1.17) | 0.37 | 1.02 (0.89 - 1.16) | 0.77 | 1.04 (0.90 - 1.19) | 0.59 |
| Insomnia | 0.98 (0.85 - 1.12) | 0.77 | 0.97 (0.84 - 1.13) | 0.72 | 0.94 (0.79 - 1.13) | 0.53 | 0.93 (0.77 - 1.12) | 0.42 |
| Snoring | 1.02 (0.87 - 1.20) | 0.76 | 1.05 (0.90 - 1.24) | 0.53 | 1.04 (0.85 - 1.28) | 0.71 | 1.04 (0.85 - 1.28) | 0.71 |
| Daytime sleepiness | 1.05 (0.90 - 1.21) | 0.55 | 1.07 (0.92 - 1.24) | 0.37 | 1.18 (0.98 - 1.43) | 0.08 | 1.17 (0.97 - 1.42) | 0.10 |
| Daytime napping | **1.13 (1.03 - 1.25)** | **0.01** | **1.15 (1.04 - 1.28)** | **0.01** | **1.19 (1.05 - 1.36)** | **0.01** | **1.19 (1.04 - 1.35)** | **0.01** |
| Sleep health score | 1.02 (0.87 - 1.20) | 0.77 | 0.99 (0.84 - 1.17) | 0.94 | 1.00 (0.83 - 1.20) | 0.99 | 1.00 (0.82 - 1.21) | 0.97 |
| **Joint effect model** |  |  |  |  |  |  |  |  |
| **Phenotypic trait** |  |  |  |  |  |  |  |  |
| Sleep duration | 0.98 (0.87 - 1.09) | 0.66 | 0.96 (0.86 - 1.08) | 0.50 | 0.89 (0.77 - 1.03) | 0.11 | 0.90 (0.77 - 1.05) | 0.17 |
| Chronotype | 1.01 (0.94 - 1.09) | 0.73 | 1.01 (0.93 - 1.09) | 0.87 | 0.99 (0.89 - 1.09) | 0.78 | 0.99 (0.89 - 1.09) | 0.79 |
| Insomnia | 1.36 (0.97 - 1.91) | 0.07 | **1.44 (1.02 - 2.03)** | **0.04** | 1.40 (0.88 - 2.25) | 0.16 | 1.37 (0.83 - 2.26) | 0.22 |
| Snoring | 1.03 (0.74 - 1.41) | 0.88 | 0.99 (0.71 - 1.37) | 0.94 | 1.00 (0.66 - 1.50) | 1.00 | 1.08 (0.70 - 1.67) | 0.73 |
| Daytime sleepiness | 1.09 (0.87 - 1.37) | 0.47 | 1.11 (0.88 - 1.40) | 0.38 | 1.23 (0.89 - 1.69) | 0.21 | 1.23 (0.89 - 1.69) | 0.21 |
| Daytime napping | 1.09 (0.89 - 1.34) | 0.42 | 1.08 (0.87 - 1.34) | 0.47 | 1.05 (0.81 - 1.37) | 0.72 | 1.07 (0.81 - 1.40) | 0.65 |
| Sleep health score | 0.96 (0.82 - 1.12) | 0.61 | 0.95 (0.82 - 1.11) | 0.55 | 0.99 (0.80 - 1.23) | 0.94 | 0.97 (0.78 - 1.21) | 0.79 |
| **Polygenic score (z-score)** |  |  |  |  |  |  |  |  |
| Sleep duration | 1.01 (0.90 - 1.12) | 0.91 | 1.03 (0.92 - 1.15) | 0.63 | 1.10 (0.96 - 1.26) | 0.16 | 1.12 (0.97 - 1.29) | 0.11 |
| Chronotype | 1.08 (0.96 - 1.20) | 0.19 | 1.05 (0.94 - 1.17) | 0.42 | 1.03 (0.89 - 1.18) | 0.73 | 1.04 (0.90 - 1.21) | 0.56 |
| Insomnia | 0.98 (0.85 - 1.12) | 0.73 | 0.97 (0.84 - 1.12) | 0.69 | 0.94 (0.78 - 1.12) | 0.48 | 0.92 (0.76 - 1.11) | 0.39 |
| Snoring | 1.02 (0.87 - 1.20) | 0.78 | 1.05 (0.90 - 1.24) | 0.52 | 1.04 (0.85 - 1.28) | 0.71 | 1.04 (0.84 - 1.27) | 0.73 |
| Daytime sleepiness | 1.05 (0.90 - 1.21) | 0.55 | 1.07 (0.92 - 1.24) | 0.37 | 1.18 (0.97 - 1.43) | 0.09 | 1.17 (0.96 - 1.41) | 0.12 |
| Daytime napping | **1.13 (1.02 - 1.24)** | **0.02** | **1.15 (1.04 - 1.27)** | **0.01** | **1.19 (1.04 - 1.35)** | **0.01** | **1.18 (1.04 - 1.35)** | **0.01** |
| Sleep health score | 1.03 (0.88 - 1.21) | 0.74 | 1.00 (0.85 - 1.18) | 0.99 | 1.00 (0.83 - 1.21) | 1.00 | 1.00 (0.82 - 1.21) | 1.00 |

**Bold** cells denote P<0.05.

^1^For independent association of phenotypic sleep traits, model was adjusted only for sex.

^2^Sociodemographic factors include education, occupational status, and marital status.

^3^Behavioral factors consist of alcohol risk consumption, physical activity, and smoking status.

^4^Health-related factors include current depressive symptomatology, use of hypnotics and sedatives, body mass index, prevalent diabetes, and prevalent cardiovascular diseases.

**eTable 20.** Main and interactive effect terms of phenotypic sleep traits and their corresponding PGS with dementia risk excluding dementia cases occurring within the first 10 years of follow-up using competing-risk analyses.

|  | **Adjusted for sex and study^1^** | | **Additionally Adjusted for Sociodemographic Factors^2^** | | **Additionally Adjusted for Behavioral Factors^3^** | | **Additionally Adjusted for Health-Related Factors^4^** | |
| --- | --- | --- | --- | --- | --- | --- | --- | --- |
|  | **HR (95% CI)** | **P** | **HR (95% CI)** | **P** | **HR (95% CI)** | **P** | **HR (95% CI)** | **P** |
| **Sleep duration** |  |  |  |  |  |  |  |  |
| **Main effect** |  |  |  |  |  |  |  |  |
| Phenotypic | 0.98 (0.87 - 1.09) | 0.67 | 0.96 (0.86 - 1.08) | 0.51 | 0.89 (0.77 - 1.03) | 0.11 | 0.90 (0.77 - 1.05) | 0.18 |
| PGS | 1.13 (0.44 - 2.95) | 0.80 | 1.13 (0.42 - 3.02) | 0.81 | 1.13 (0.40 - 4.15) | 0.68 | 1.74 (0.48 - 6.33) | 0.40 |
| **Interaction term** |  |  |  |  |  |  |  |  |
| Phenotypic*PGS | 0.99 (0.88 - 1.10) | 0.81 | 0.99 (0.88 - 1.11) | 0.85 | 0.98 (0.85 - 1.13) | 0.79 | 0.95 (0.81 - 1.10) | 0.50 |
| **Chronotype** |  |  |  |  |  |  |  |  |
| **Main effect** |  |  |  |  |  |  |  |  |
| Phenotypic | 1.01 (0.94 - 1.09) | 0.76 | 1.00 (0.93 - 1.09) | 0.92 | 0.98 (0.89 - 1.08) | 0.72 | 0.98 (0.89 - 1.09) | 0.76 |
| PGS | 1.09 (0.97 - 1.22) | 0.16 | 1.06 (0.94 - 1.20) | 0.34 | 1.06 (0.90 - 1.20) | 0.62 | 1.05 (0.90 - 1.22) | 0.52 |
| **Interaction term** |  |  |  |  |  |  |  |  |
| Phenotypic*PGS | 0.98 (0.91 - 1.05) | 0.54 | 0.97 (0.90 - 1.05) | 0.49 | 0.97 (0.88 - 1.07) | 0.51 | 0.98 (0.89 - 1.08) | 0.68 |
| **Insomnia** |  |  |  |  |  |  |  |  |
| **Main effect** |  |  |  |  |  |  |  |  |
| Phenotypic | 1.36 (0.97 - 1.91) | 0.07 | 1.44 (1.02 - 2.03) | 0.04 | 1.41 (0.88 - 2.26) | 0.16 | 1.38 (0.83 - 2.27) | 0.21 |
| PGS | 0.96 (0.82 - 1.13) | 0.63 | 0.94 (0.80 - 1.12) | 0.49 | 0.94 (0.74 - 1.14) | 0.45 | 0.90 (0.72 - 1.13) | 0.38 |
| **Interaction term** |  |  |  |  |  |  |  |  |
| Phenotypic*PGS | 1.07 (0.80 - 1.43) | 0.66 | 1.13 (0.84 - 1.52) | 0.41 | 1.09 (0.78 - 1.53) | 0.62 | 1.08 (0.76 - 1.55) | 0.66 |
| **Snoring** |  |  |  |  |  |  |  |  |
| **Main effect** |  |  |  |  |  |  |  |  |
| Phenotypic | 1.03 (0.74 - 1.42) | 0.87 | 0.99 (0.71 - 1.37) | 0.94 | 1.00 (0.66 - 1.51) | 0.99 | 1.08 (0.69 - 1.68) | 0.74 |
| PGS | 0.99 (0.80 - 1.23) | 0.94 | 1.00 (0.80 - 1.24) | 0.99 | 1.00 (0.68 - 1.28) | 0.68 | 0.93 (0.68 - 1.27) | 0.65 |
| **Interaction term** |  |  |  |  |  |  |  |  |
| Phenotypic*PGS | 1.07 (0.78 - 1.46) | 0.69 | 1.12 (0.82 - 1.53) | 0.49 | 1.20 (0.79 - 1.84) | 0.39 | 1.21 (0.79 - 1.86) | 0.39 |
| **Daytime sleepiness** |  |  |  |  |  |  |  |  |
| **Main effect** |  |  |  |  |  |  |  |  |
| Phenotypic | 1.04 (0.83 - 1.31) | 0.72 | 1.07 (0.85 - 1.35) | 0.56 | 1.17 (0.84 - 1.62) | 0.36 | 1.17 (0.84 - 1.63) | 0.36 |
| PGS | 0.77 (0.55 - 1.08) | 0.13 | 0.84 (0.60 - 1.19) | 0.33 | 0.84 (0.64 - 1.47) | 0.90 | 0.99 (0.65 - 1.51) | 0.97 |
| **Interaction term** |  |  |  |  |  |  |  |  |
| Phenotypic*PGS | **1.25 (1.01 - 1.55)** | **0.04** | 1.19 (0.96 - 1.49) | 0.12 | 1.15 (0.90 - 1.48) | 0.26 | 1.13 (0.87 - 1.46) | 0.35 |
| **Daytime napping** |  |  |  |  |  |  |  |  |
| **Main effect** |  |  |  |  |  |  |  |  |
| Phenotypic | 1.07 (0.87 - 1.32) | 0.52 | 1.06 (0.85 - 1.32) | 0.60 | 1.02 (0.77 - 1.34) | 0.89 | 1.04 (0.78 - 1.38) | 0.78 |
| PGS | 1.07 (0.94 - 1.23) | 0.30 | 1.09 (0.94 - 1.25) | 0.25 | 1.09 (0.93 - 1.32) | 0.25 | 1.11 (0.93 - 1.33) | 0.24 |
| **Interaction term** |  |  |  |  |  |  |  |  |
| Phenotypic*PGS | 1.11 (0.91 - 1.35) | 0.31 | 1.13 (0.92 - 1.38) | 0.25 | 1.16 (0.90 - 1.49) | 0.25 | 1.14 (0.88 - 1.47) | 0.32 |
| **Sleep Health Score** |  |  |  |  |  |  |  |  |
| **Main effect** |  |  |  |  |  |  |  |  |
| Phenotypic | 0.96 (0.82 - 1.12) | 0.59 | 0.95 (0.82 - 1.11) | 0.54 | 0.99 (0.80 - 1.23) | 0.94 | 0.97 (0.78 - 1.21) | 0.80 |
| PGS | 0.89 (0.54 - 1.47) | 0.65 | 0.93 (0.56 - 1.55) | 0.79 | 0.93 (0.59 - 2.02) | 0.78 | 1.04 (0.55 - 1.95) | 0.91 |
| **Interaction term** |  |  |  |  |  |  |  |  |
| Phenotypic*PGS | 1.05 (0.89 - 1.23) | 0.56 | 1.02 (0.87 - 1.20) | 0.78 | 0.97 (0.79 - 1.19) | 0.78 | 0.99 (0.80 - 1.23) | 0.91 |

**Bold** cells denote P<0.05.

^1^For independent association of phenotypic sleep traits, model was adjusted only for sex.

^2^Sociodemographic factors include education, occupational status, and marital status.

^3^Behavioral factors consist of alcohol risk consumption, physical activity, and smoking status.

^4^Health-related factors include current depressive symptomatology, use of hypnotics and sedatives, body mass index, prevalent diabetes, and prevalent cardiovascular diseases.

# eDiscussion

In analyses that did not exclude dementia diagnoses occurring within the first 10 years of follow-up, insomnia was associated with a lower risk of dementia, and the association remained comparable within twin-pair analysis, indicating that shared genetic or familial factors are unlikely to account for the association. Recent pooled analyses from multiple studies^13,14^ found that insomnia is associated with an elevated risk of all-cause dementia, although substantial heterogeneity was observed across studies due to age, definition of insomnia, and study design. Using Mendelian randomization (MR) in a large-scale genetic analysis, Andrews and colleagues^15^ found no evidence of a causal relationship between insomnia symptoms and increased risk of AD, nor with core neuropathological markers such as amyloid plaques and tau tangles, when assessed using genetic proxies. Contrary to expectations, an inverse association between insomnia and dementia was observed in our study, but this disappeared once reverse causation was taken into account. Prior work suggests that studies with shorter follow-up periods may be particularly vulnerable to reverse causation because prodromal neurodegenerative changes can alter sleep patterns.^16^ This issue is especially relevant in older adults, who are more likely to be in the prodromal dementia phase. In our younger older-adult subsample with both phenotypic and genotypic data, insomnia was not associated with dementia, regardless of whether reverse causation was considered.

Longer sleep duration was associated with lower dementia risk once early dementia diagnoses were excluded. Meta-analyses report a U-shaped association between sleep duration and dementia, with both short and excessively long sleep linked to higher risk and the lowest risk observed around 7 hours.^17,18^ However, many of these studies had follow-up periods under 10 years and are therefore vulnerable to reverse causation, given the long preclinical phase of dementia. In contrast, a long-term cohort with ⁓25 years of follow-up found that short sleep (≤6 hours vs. 7 hours) in midlife predicted increased dementia risk, while no evidence was found with long sleep duration (≥8 hours vs. 7 hours).^19^ Another meta-analysis showed that short sleep duration increased dementia risk only in studies with short follow-up, whereas long sleep was associated with elevated risk regardless of follow-up duration, though results were heterogenous and of mixed quality. ^20^

We observed no association between the PGS for midlife sleep duration and dementia. This may reflect that overall sleep duration is not the most sensitive sleep-related predictor of dementia risk. Prior work in the SALT cohort showed that short sleep, extended time in bed and delayed rising were associated with dementia, using composite indices from the Karolinska Sleep Questionnaire,^21^ whereas our analyses focused on individual sleep items and harmonized definitions with the UK Biobank to ensure PGS compatibility. Using short or long sleep categories would have substantially reduced sample size, as few participants met the thresholds in the combined sample. A recent meta-analysis also reported that shorter sleep duration is associated with greater amyloid-beta burden on positron emission tomography imaging, supporting its potential relevance to AD pathology.^22^ Further analyses using genetically predicted short or long sleep duration may help clarify their specific contribution to dementia risk.

# eReferences

1.	Choi SW, Mak TS, O'Reilly PF. Tutorial: a guide to performing polygenic risk score analyses. *Nat Protoc*. Sep 2020;15(9):2759-2772. doi:10.1038/s41596-020-0353-1

2.	Lloyd-Jones LR, Zeng J, Sidorenko J, et al. Improved polygenic prediction by Bayesian multiple regression on summary statistics. *Nature communications*. Nov 8 2019;10(1):5086. doi:10.1038/s41467-019-12653-0

3.	Nordin M, Åkerstedt T, Nordin S. Psychometric evaluation and normative data for the Karolinska Sleep Questionnaire: Evaluation of the KSQ. *Sleep and biological rhythms*. 2013;11(4):216-226. doi:10.1111/sbr.12024

4.	Rizzuto D, Feldman AL, Karlsson IK, Dahl Aslan AK, Gatz M, Pedersen NL. Detection of Dementia Cases in Two Swedish Health Registers: A Validation Study. *J Alzheimers Dis*. 2018;61(4):1301-1310. doi:10.3233/jad-170572

5.	Stunkard AJ, Harris JR, Pedersen NL, McClearn GE. The body-mass index of twins who have been reared apart. *N Engl J Med*. May 24 1990;322(21):1483-7. doi:10.1056/nejm199005243222102

6.	Dashti HS, Jones SE, Wood AR, et al. Genome-wide association study identifies genetic loci for self-reported habitual sleep duration supported by accelerometer-derived estimates. *Nature communications*. Mar 7 2019;10(1):1100. doi:10.1038/s41467-019-08917-4

7.	Campos AI, García-Marín LM, Byrne EM, Martin NG, Cuéllar-Partida G, Rentería ME. Insights into the aetiology of snoring from observational and genetic investigations in the UK Biobank. *Nature communications*. 2020/02/14 2020;11(1):817. doi:10.1038/s41467-020-14625-1

8.	Wang H, Lane JM, Jones SE, et al. Genome-wide association analysis of self-reported daytime sleepiness identifies 42 loci that suggest biological subtypes. *Nature communications*. 2019/08/13 2019;10(1):3503. doi:10.1038/s41467-019-11456-7

9.	Jones SE, Lane JM, Wood AR, et al. Genome-wide association analyses of chronotype in 697,828 individuals provides insights into circadian rhythms. *Nature communications*. 2019/01/29 2019;10(1):343. doi:10.1038/s41467-018-08259-7

10.	Lane JM, Jones SE, Dashti HS, et al. Biological and clinical insights from genetics of insomnia symptoms. *Nat Genet*. Mar 2019;51(3):387-393. doi:10.1038/s41588-019-0361-7

11.	Goodman MO, Faquih T, Paz V, et al. Genome-wide association analysis of composite sleep health scores in 413,904 individuals. *Communications Biology*. 2025/01/24 2025;8(1):115. doi:10.1038/s42003-025-07514-0

12.	Dashti HS, Daghlas I, Lane JM, et al. Genetic determinants of daytime napping and effects on cardiometabolic health. *Nature communications*. Feb 10 2021;12(1):900. doi:10.1038/s41467-020-20585-3

13.	Ungvari Z, Fekete M, Lehoczki A, et al. Sleep disorders increase the risk of dementia, Alzheimer's disease, and cognitive decline: a meta-analysis. *Geroscience*. Jun 2025;47(3):4899-4920. doi:10.1007/s11357-025-01637-2

14.	Meng M, Shen X, Xie Y, Lan R, Zhu S. Insomnia and risk of all-cause dementia: A systematic review and meta-analysis. *PloS one*. 2025;20(4):e0318814. doi:10.1371/journal.pone.0318814

15.	Andrews SJ, Fulton-Howard B, O'Reilly P, Marcora E, Goate AM. Causal Associations Between Modifiable Risk Factors and the Alzheimer's Phenome. *Ann Neurol*. Jan 2021;89(1):54-65. doi:10.1002/ana.25918

16.	Alders P, Kok A, van Zutphen EM, Claassen J, Deeg DJH. The effect of sleep disturbances on the incidence of dementia for varying lag times. *J Prev Alzheimers Dis*. Feb 2025;12(2):100024. doi:10.1016/j.tjpad.2024.100024

17.	Fan L, Xu W, Cai Y, Hu Y, Wu C. Sleep Duration and the Risk of Dementia: A Systematic Review and Meta-analysis of Prospective Cohort Studies. *Journal of the American Medical Directors Association*. 2019;20(12):1480-1487.e5. doi:10.1016/j.jamda.2019.06.009

18.	Liang Y, Qu L-B, Liu H. Non-linear associations between sleep duration and the risks of mild cognitive impairment/dementia and cognitive decline: a dose–response meta-analysis of observational studies. *Aging Clinical and Experimental Research*. 2019/03/01 2019;31(3):309-320. doi:10.1007/s40520-018-1005-y

19.	Sabia S, Fayosse A, Dumurgier J, et al. Association of sleep duration in middle and old age with incidence of dementia. *Nature communications*. Apr 20 2021;12(1):2289. doi:10.1038/s41467-021-22354-2

20.	Howard C, Mukadam N, Hui EK, Livingston G. The effects of sleep duration on the risk of dementia incidence in short and long follow-up studies: A systematic review and meta-analysis. *Sleep medicine*. 2024/12/01/ 2024;124:522-530. doi:<https://doi.org/10.1016/j.sleep.2024.10.022>

21.	Bokenberger K, Ström P, Dahl Aslan AK, et al. Association Between Sleep Characteristics and Incident Dementia Accounting for Baseline Cognitive Status: A Prospective Population-Based Study. *The journals of gerontology Series A, Biological sciences and medical sciences*. Jan 2017;72(1):134-139. doi:10.1093/gerona/glw127

22.	Chen C-L, Zhang M-Y, Wang Z-L, et al. Associations among sleep quality, sleep duration, and Alzheimer's disease biomarkers: A systematic review and meta-analysis. *Alzheimer's & Dementia*. 2025;21(3):e70096. doi:<https://doi.org/10.1002/alz.70096>
